# Deriving the anticholinergic medication index for delirium and falls in older people: development and external validation of a risk prediction model

**DOI:** 10.1136/bmjmed-2026-002835

**Published:** 2026-11-04

**Authors:** Kate E Best, Olly Butters, Pieta Georgina Schofield, Oliver Todd, Sarah Alderson, David P Alldred, Laura Bonnett, Iain Buchan, Amanda Farrin, Owen Johnson, Ciarán McInerney, David Mehdizadeh, Tom Lawton, Rebecca Lawton, Farag Shuweidhi, Lauren E Walker, Robert West, Bridget Young, Munir Pirmohamed, Bethan Copsey, Aseel S Abuzour, Sarah E Rodgers, Andrew Clegg

**Affiliations:** 1 Academic Unit for Ageing and Stroke Research, Leeds Institute of Health Sciences, University of Leeds, Leeds, UK; 2 Bradford Institute for Health Research, Bradford Teaching Hospitals NHS Foundation Trust, Bradford, UK; 3 Public Health, Policy and Systems, Institute of Population Health, University of Liverpool, Liverpool, UK; 4 Leeds Institute of Health Sciences, University of Leeds, Leeds, UK; 5 School of Healthcare, University of Leeds, Leeds, UK; 6 NIHR Yorkshire and Humber Patient Safety Research Collaboration, Leeds, UK; 7 Institute of Population Health, University of Liverpool, Liverpool, UK; 8 Leeds Institute of Clinical Trials Research, University of Leeds, Leeds, UK; 9 Edinburgh Clinical Trials Unit, University of Edinburgh, Edinburgh, UK; 10 School of Computer Science, University of Leeds, Leeds, UK; 11 School of Medicine and Population Health, University of Sheffield, Sheffield, UK; 12 Improvement Academy, Bradford Teaching Hospitals NHS Foundation Trust, Bradford, UK; 13 School of Psychology, University of Leeds, Leeds, UK; 14 Centre for Experimental Therapeutics, Institute of Systems, Molecular, and Integrative Biology, University of Liverpool, Liverpool, UK; 15 Wolfson Centre for Personalised Medicine, Institute of Systems, Molecular, and Integrative Biology, University of Liverpool, Liverpool, UK

**Keywords:** Health services, Medicine, Primary health care

## Abstract

**Objective:**

To develop the anticholinergic medication index (ACMI), a new anticholinergic burden measure derived from a clinical prediction model for falls and delirium, scoring 88 anticholinergic medicines prescribed in primary care.

**Design:**

Development and external validation of a risk prediction model.

**Setting:**

UK primary and secondary care data from Connected Bradford and the Secure Anonymised Information Linkage (SAIL) databank, 1 January 2019 to 31 December 2019.

**Participants:**

For development cohort, 71 757 Bradford and Craven residents aged >65 years on 1 January 2019 who contributed data to Connected Bradford between 1 January 2019 and 31 December 2019. For external validation cohort, 678 134 Welsh residents aged >65 years on 1 January 2019 who contributed data to SAIL between 1 January 2019 and 31 December 2019.

**Main outcome measures:**

Composite outcome of hospital admission for delirium or hospital admission for a fall or fracture (as an indicator of an injurious fall) during the study period.

**Results:**

71 757 patients (4147 (5.8%) with the composite outcome) comprised the development cohort and 678 134 patients (43 675 (6.4%) with the composite outcome) comprised the external validation cohort. Based on anticholinergic medications recorded in electronic health records as predictors, a prediction model for falls and delirium was developed and validated. Model coefficients for anticholinergic medications were transformed into ACMI scores. Of 88 anticholinergic medications, codeine, prednisolone, and furosemide were the most commonly prescribed (8.3%, 6.5%, and 6.0% of patients prescribed these anticholinergic drugs at least once in the development cohort, respectively). Model discrimination and calibration in the development cohort was good (C index=0.794, R^2^=0.06, calibration slope=0.97). The model performed well in external validation with good discrimination (C index=0.721, 95% confidence interval (CI) 0.718 to 0.734, R^2^=0.04) but with some evidence of miscalibration (calibration slope=0.69). The ACMI medications were scored 0.1 (desloratadine) to 2.3 (amantadine).

**Conclusions:**

The ACMI was designed to be implemented in primary care to automatically calculate anticholinergic burden. Combined with the model predicted risk of delirium or falls, the ACMI allows clinicians to prioritise patients for structured medication reviews to reduce overprescribing.

**WHAT IS ALREADY KNOWN ON THIS TOPIC**

- Physiological changes that occur with age mean that older people are susceptible to adverse outcomes associated with anticholinergic medicines
- Structured medication reviews can reduce overprescribing of potentially inappropriate medications and therefore the risk of adverse outcomes
- Multiple tools are available to help practitioners estimate anticholinergic drug burden, but the weighting of each medication is typically based on drug potency rather than prediction of adverse outcomes, and the tools are not integrated into electronic health record systems

**WHAT THIS STUDY ADDS**

- Based on anticholinergic medications recorded in electronic health records as predictors, a prediction model for falls and delirium was developed and validated
- An anticholinergic medication index (ACMI) was derived, scoring 88 anticholinergic medications from 0.1 to 2.3 based on the regression coefficients of the prediction model

**HOW THIS STUDY MIGHT AFFECT RESEARCH, PRACTICE OR POLICY**

- The ACMI can be implemented in primary care systems to provide a simple way for clinicians to prioritise patients who would benefit most from a structured medication review
- The ACMI enables clinicians to efficiently access a patient's anticholinergic burden, which can help inform prescribing decisions

## Introduction

 Anticholinergic medicines block the neurotransmitter acetylcholine in the brain and peripheral nervous system.^1^ In some instances, the anticholinergic effect is the intended mechanism of action (eg, antimuscarinic drugs for an overactive bladder and intestinal spasm), but in others, the anticholinergic effect is a secondary effect (eg, antidepressants and antihistamines). More than 100 medications with anticholinergic properties are prescribed in the UK and USA,^2,3^ with up to 90% of older adults prescribed at least one annually in the UK and Ireland.^3–5^ Anticholinergic burden is the cumulative effect of taking multiple anticholinergic medications.

Prescribing of anticholinergic medications is often necessary and the benefits can outweigh the harms. But because of physiological changes with ageing, older people are susceptible to the adverse effects of anticholinergic medications, with a higher burden associated with an increased risk of falls and delirium (acute confusion) and decreased cognitive function.^6–8^ These events are common reasons for hospital admission and have a major impact on the health and wellbeing of older people, their families, and carers, with considerable cost to the health and social care system. This situation is particularly problematic given the ninefold increase in the anticholinergic burden between 1990 and 2015 in the UK and the lack of a reduction in anticholinergic prescribing in the US during 2006-15.^3,9^ Anticholinergic medicines represent a potentially modifiable contributor to adverse outcomes but are often overlooked in routine practice, with additional evidence from clinical trials indicating that not reducing the anticholinergic burden could explain the lack of impact on clinical outcomes.^10^ Gaps in training on anticholinergic medications and the absence of accessible clinical decision support systems are recognised as potentially contributory factors.^10^

Consensus is lacking on scoring the anticholinergic burden, with multiple scales available.^2^ These scales assign points to anticholinergic medications, with the anticholinergic burden being the sum of the points of the concurrently prescribed medications. In each scale, the medications included and the points assigned vary,^2^ but both are usually derived by expert consensus on biological potency, rather than on how well they predict well defined adverse clinical outcomes, which may improve clinical relevance.^11^ The anticholinergic cognitive burden scale, which includes 88 medications scored 1-3, has been validated in different healthcare settings^12^ and is included in the National Institute for Health and Care Excellence (NICE) dementia guidance.^13^ The requirement for the anticholinergic cognitive burden score to be calculated manually or from data input into a website^14^ by clinicians, however, is a barrier to translating this tool into routine care. The widespread availability of primary and secondary care electronic health record systems could support more complex, automated calculations of anticholinergic burden.

The 2023 chief medical officer's annual report, Health in an Ageing Society, recommended a structured approach to prescribing and reviewing medicines to reduce the risk of harm.^15^ NICE guidelines recommend targeted medication reviews as part of inpatient delirium and prevention of falls.^16,17^ Primary care networks in England are required to target patients with problematic polypharmacy through a structured medication review. An anticholinergic burden score that can be derived automatically from electronic health records could enable clinicians to group patients at highest risk of adverse events, enabling prioritisation of structured medication reviews.

The aim of this study was to derive a new measure of anticholinergic burden, the anticholinergic medication index (ACMI). The weights of each medication in the ACMI are transformations of coefficients from the development and external validation of a new clinical prediction model for falls and delirium. A core aim of the study was to develop the ACMI for transferability within the UK and internationally, ensuring that the index would be suitable for future use across available electronic health record systems to help clinicians group patients by risk of who would benefit from medication reviews.

## Methods

Figure 1 shows the development and external validation process of the ACMI.

**Figure 1:**
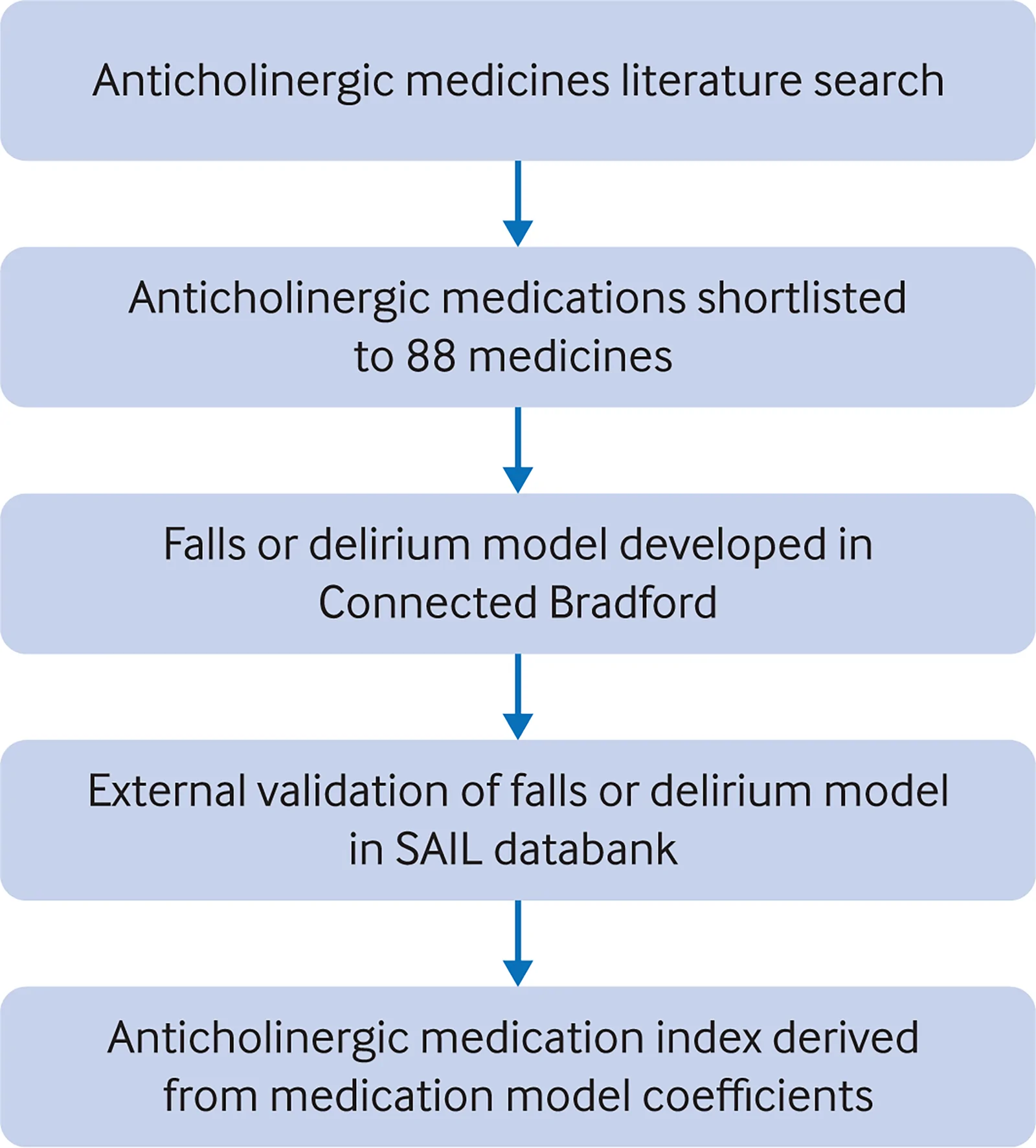
Flow diagram showing the process of developing the anticholinergic medication index. SAIL=Secure Anonymised Information Linkage

### Study design

A retrospective cohort study based on routinely recorded data from Connected Bradford (www.bradfordresearch.nhs.uk/connected-bradford/)^18^ was used to develop a clinical prediction model that predicted the one year risk of hospital admission with delirium or with a serious fall (defined as hospital admission with a fall or fracture). External validation of the prediction model was performed in a second retrospective cohort from the Secure Anonymised Information Linkage (SAIL) databank (www.saildatabank.com/).^19^ Although secondary care data were used to determine outcomes, only primary care data were used to determine predictors, to ensure that the model could be implemented in primary care electronic health record systems. The ACMI was derived from the clinical prediction model, by using the transformed model coefficients to assign weights to each anticholinergic medication.

### Data sources

Connected Bradford consists of anonymised linked primary, secondary, and social care data on patients who received care in Bradford and Craven, UK. The Bradford district and Craven local authority area includes about 600 000 people, with services from five NHS trusts and 86 general practices.^18^ Bradford is a post-industrial city in the North of England. About 30% of the Bradford population are of South Asian origin (predominantly Pakistani) with the district being the fifth most income deprived local authority in England.^20–22^ In 2018-19, 23% and 9% of the population aged >65 years were in English index of multiple deprivation groups 1 and 5, respectively (where the index of multiple deprivation is categorised as five equal groups (groups 1-5), with group 1 being the most deprived and group 5 the least deprived group).^23^ The SAIL databank has anonymised records from about 2.7 million patients registered with participating general practices in Wales (85% of the population of Wales), including linked primary care data, emergency department attendance, hospital admissions, and mortality data from the Office for National Statistics.^19^ The ethnic population of Wales is 93.8% white and 2.9% Asian, and income deprivation is similar to the average in England.^24^ In 2018-19, 13% and 19% of the population aged >65 years were in Welsh index of multiple deprivation group 1 (most deprived) and group 5 (least deprived), respectively.^23^

### Participants

For the development cohort, Bradford and Craven residents aged >65 years on 1 January 2019 who contributed data to Connected Bradford between 1 January 2019 and 31 December 2019 were included. The same criteria were used to select Welsh residents as part of the SAIL databank for the external validation cohort. People aged >99 years were excluded in both cohorts to limit potential patient uniqueness, thereby reducing the risk of re-identification.

### Outcomes

Falls and delirium have the strongest mechanistic links to anticholinergic medications,^7,25^ and are of considerable importance to patients, carers, and health and social care systems, costing £2.3bn (€2.7bn; US$3.1bn) and £10.8bn to the NHS annually, respectively.^26,27^ In this study, the outcome was a composite of hospital admission for delirium or hospital admission for a fall or fracture (as an indicator of an injurious fall) during the study period, defined as 1 January 2019 to 31 December 2019. After careful consideration, a composite outcome was used because the intervention (structured medication review) offered to those at high risk of either outcome would be the same, and deriving only one ACMI score was more practical. Systemised Nomenclature of Medicine-Clinical Terms (SNOMED CT) codes for delirium were identified by key word searching in Athena, the Observational Health Data Sciences and Informatics Vocabularies Repository,^28^ screened by clinical coauthor AC for use in Connected Bradford. These codes were mapped to ICD-10 (international classification of diseases, 10th revision) codes for use in the external validation. SNOMED CT and ICD-10 code lists for fall and fracture were those validated in recent research.^23^ Code lists are available from https://github.com/ConnectedBradford/CB_1322_ACMI_development.

### Anticholinergic medications

Candidate anticholinergic medications were identified from existing international scales and based on international non-proprietary names to support transferability.^29–31^ Pharmacologist LEW and pharmacist DM independently screened the candidate medications to exclude those not routinely prescribed in the UK and those with no evidence of anticholinergic effect, and together with coauthors with pharmacological expertise (MP, AC, and DPA), reached a consensus on 109 medications. Anticholinergic medications with a prescribing rate <0.1% in England (n=11) during March 2021 according to OpenPrescribing.net,^32^ and medications associated with ≤1 outcome (n=10) during the study period, were further excluded (to enable model convergence), resulting in 88 candidate medications.

SNOMED CT and Dictionary of Medicines and Devices codes for the selected anticholinergic medications, including brand names and compounds, were extracted from the NHS Business Services Authority coding list,^33^ resulting in 21 505 codes, available from the corresponding author on request. Topical treatments were identified by text screening the product descriptions from the Dictionary of Medicines and Devices (keywords ointment, gel, and cream) and were excluded because of limited systemic use. Medications prescribed to be taken as required were also excluded because the use period was considered potentially highly variable. In the development cohort, as required medications were identified by text searching key phrases (eg, PRN and as required) in the free text prescription signature. As required medications were included in the external validation cohort because the prescription signature was not accessible in SAIL data.

In the development cohort, the drug use period was calculated from the start and end dates of the prescription. In the external validation cohort, prescription end dates were not provided and therefore the drug use period was classed as 30 days from the start date of the prescription, to reflect the recommended prescribing interval in Wales.^34^

### Predictors

Model predictors included 88 binary time varying covariates to represent the monthly drug use status of each of the 88 anticholinergic medications during the one year study period (eg, separate drug use statuses in January 2019, February 2019, and December 2019). When using time varying covariates, use of the drug at month t was used to estimate the risk of the outcome by time t (ie, month 12 drug use status was used to predict the one year risk). Given the short washout period of anticholinergic medications, use of the drug at the time of the outcome (month 12) was considered more relevant than drug use at, for example, study entry (month 1) in the model estimation. For many patients, however, drug use status remained relatively stable because the medications were frequently repeat prescriptions. Use of each medication was coded as binary (used or not used in each month). After discussions with suppliers of primary care electronic health record systems, all forms of medication (eg, oral solution or tablet) were combined, and drug dose was not represented in the model, because accurately estimating drug dose in electronic health record systems is challenging. This approach ensured a final model suitable for widespread use across electronic health record systems, minimising the potential for research waste.

Other predictors were key predictors of injurious falls and delirium recorded on or before the start of the study period (1 January 2019): age at study entry (continuous, centred at the median), sex (male or female), cognitive impairment or dementia, visual impairment, hearing impairment, incontinence, osteoporosis, history of falls or fragility fracture, parkinsonism or tremor, arthritis (all coded present or absent), and the electronic frailty index score (continuous, centred at the median).^35^ Data for sex were taken from information in Connected Bradford and SAIL and might not correspond to patient reported gender. The electronic frailty index is calculated as a proportion of 36 deficits (eg, diabetes, anaemia, and asthma) present in a patient. The score ranges from 0 to 1, with a higher score indicating greater frailty. Similar electronic frailty index scores have been validated internationally.^36–38^ In this study, the electronic frailty index was adapted to be calculated based on 28 deficits (36 minus the eight deficits individually incorporated into the model as predictors). SNOMED CT codes were taken from previously validated lists^35^ with any diagnosis over the life course before the study start date included. The SNOMED CT codes were mapped to Read codes version 2, with standard mapping tables to enable validation in SAIL. We included anticholinergic medications and comorbidities recorded in primary care only, because this information will be available in the intended setting for implementation.

### Statistical analysis

A Cox model was fitted in the development cohort, adjusted for 88 time varying covariates representing the 88 anticholinergic medications, and the additional predictors, with hospital admission for delirium or falls during the 12 months of follow-up as the composite outcome. Next, the exponential of the linear predictor was multiplied by the baseline hazard (development cohort) and applied to the data in the external validation cohort. The linear predictor was estimated with the use of anticholinergic agents in month 1 and then, for comparison, in month 12.

Model fit statistics (bias corrected: Harrell's C index, Nagelkerke R^2^, observed/expected, and calibration slope) were produced for the clinical prediction model in both cohorts and interpreted following best practice on evaluation of prediction models.^39^ Fivefold cross validation was applied for interval validation, meaning the data are split randomly into five folds with the model being trained on four folds and tested on the remaining one. The process is repeated five times and model fit statistics are then averaged over the five iterations. Calibration plots showing the predicted versus observed risk of admission to hospital for falls or delirium at month 12, based on anticholinergic drug use status at month 1, were fitted in the development and external validation cohorts. Although drug use status at month 12 would be the more accurate estimate, when the ACMI is implemented in practice, only the current use of anticholinergic agents (ie, month 1) would be known. Calibration according to the Welsh index of multiple deprivation groups (categorised as five equal groups (groups 1-5), with group 1 being the most deprived and group 5 the least deprived group) was also examined in the external validation cohort.

### Calculating the anticholinergic medication index

To align the ACMI tool with similar scales, we transformed the anticholinergic medication coefficients from the clinical prediction model to derive the ACMI. We shifted the values of the coefficients to a positive scale by adding the modulus of the value of the lowest negative coefficient because no medicines protective of anticholinergic activity exist (lowest value=−1.4). We then calculated the percentage contribution of each shifted coefficient to the hypothetical maximum possible ACMI score (total possible=137.57), meaning a hypothetical patient prescribed all 88 anticholinergic medications would have an ACMI score of 100, whereas a patients prescribed no anticholinergic medications would have an ACMI score of 0.

### Comparison with an existing scale

A version of the anticholinergic cognitive burden score was calculated for each patient for each month in the development and external validation cohorts. Although the anticholinergic cognitive burden scale has 88 medications, only 44 were in our list of candidate medications with known anticholinergic properties prescribed in the UK. The anticholinergic cognitive burden score was entered into the falls or delirium clinical prediction model in place of the anticholinergic medications but including all of the other predictors. Discrimination statistics (C index and Nagelkerke's R^2^) were produced in fivefold cross validations for the adjusted and unadjusted versions of the model.

### Sample size

With Riley et al's pmsampsi package in Stata, we derived a prespecified sample size calculation for model development.^40^ Based on existing estimates of hospital admission for falls and delirium in the community (5% and 1-2%, respectively),^35,41,42^ we estimated that 6-7% of patients would have our composite outcome. Conservatively, based on the lower range of the estimated incidence, and assuming a maximum of 150 predictor parameters included in the final model, with R^2^ values ≤5%, we calculated that 26 243 patients were required for model development to ensure that overfitting and optimism were minimised.

Guidance on sample size for prediction models recommends tailoring the sample size required for external validation based on the model being validated.^43^ A sample size of 60 000 patients (with at least 4000 with the composite outcome) was sufficient to target a 95% confidence interval (CI) of width 0.2 around the estimate of the calibration slope. Sample sizes in the development and external validation cohorts exceeded the sample size estimates required.

### Missing data

In both datasets, patients with missing age or sex were not included and other predictors were assumed to be present if recorded in a patient's primary care record and not present if not recorded.

### Patient and public involvement

We established an ACMI patient and public involvement group through Health Data Research UK North, consisting of seven lay representatives with lived experience of taking multiple medications, including anticholinergic medications, or providing carer support for people taking multiple medications. The ACMI patient and public involvement group regularly met during the project, steering the work throughout, including identification of outcomes of importance, discussing potential predictors to be included, and emphasising the need to develop a scoring system that could be used in routine practice. The patient and public involvement group gave input into what the ACMI score should look like, how it might be interpreted by patients, and how risk should be communicated to patients. The group also emphasised the potential of an automated anticholinergic score to help deal with health inequalities. The patient and public involvement group is developing a summary document to help disseminate this work to relevant patient and public stakeholder groups.

## Results

### Study population and characteristics

We included 71 757 patients in the development cohort and 678 134 in the external validation cohort. Tables 1 and 2 present the personal characteristics of the patients, the prevalence of the predictors, and the proportions of the population prescribed each anticholinergic medication during the study period. The incidence of the composite outcome was comparable across cohorts (5.8% *v* 6.4%), although the incidence of falls was lower in the development cohort (4.3% *v* 5.7%), and the incidence of delirium was higher (2.4% *v* 1.1%).

**Table 1:** Baseline characteristics in development and external validation cohorts

Characteristics	Development cohort (n=71 757)	External validation cohort (n=678 134)
Median (IQR) age (years)	74 (70-81)	74 (70-81)
Age (years)
65-74	37 012 (51.6)	347 859 (51.3)
75-84	23 774 (33.1)	225 565 (33.3)
≥85	10 971 (15.3)	104 710 (15.4)
Women	38 653 (53.9)	359 824 (53.1)
Median (IQR) electronic frailty index score	0.07 (0.04, 0.15)	0.07 (0.04, 0.15)
Electronic frailty index frailty category*:		
Fit	47 636 (66.4)	434 328 (64.0)
Mild	17 128 (23.9)	182 150 (26.9)
Moderate	5680 (7.9)	52 497 (7.7)
Severe	1313 (1.8)	9159 (1.4)
Cognitive impairment	11 153 (15.5)	37 739 (5.6)
Visual impairment	14 398 (20.1)	166 116 (24.5)
Arthritis	27 608 (38.5)	211 323 (31.2)
History of falls	10 770 (15.0)	120 233 (17.7)
Incontinence	5603 (7.8)	41 086 (6.1)
Hearing impairment	10 396 (14.5)	135 435 (20.0)
Osteoporosis	8337 (11.6)	48 265 (7.1)
Parkinson's disease	1476 (2.1)	9861 (1.5)
Falls	3081 (4.3)	38 839 (5.7)
Delirium	1698 (2.4)	7729 (1.1)
Falls or delirium	4147 (5.8)	43 675 (6.4)

Data are number (%) unless indicated otherwise.

* Categories derived with cut-off points published elsewhere.

IQR, interquartile range.

**Table 2:** Patients prescribed anticholinergic medication at least once during the one year study period

Anticholinergic medication	Development cohort (No (%) of patients)	External validation cohort (No (%) of patients)
Codeine	5985 (8.3)	61 110 (9.0)
Prednisolone	4683 (6.5)	57 477 (8.5)
Furosemide	4274 (6.0)	61 154 (9.0)
Amitriptyline	4091 (5.7)	39 833 (5.9)
Bendroflumethiazide	3591 (5.0)	44 870 (6.6)
Indapamide	3333 (4.6)	19 159 (2.8)
Umeclidinium	2724 (3.8)	88 (0.01)
Atenolol	2596 (3.6)	23 672 (3.5)
Doxazosin	2417 (3.4)	21 747 (3.2)
Ranitidine	1870 (2.6)	24 604 (3.6)
Cetirizine	1763 (2.5)	17 439 (2.6)
Citalopram	1605 (2.2)	24 531 (3.6)
Mirtazapine	1566 (2.2)	19 018 (2.8)
Dexamethasone	1446 (2.0)	3100 (0.5)
Warfarin	1431 (2.0)	29 654 (4.4)
Tiotropium bromide	1279 (1.8)	9150 (1.4)
Solifenacin	1094 (1.5)	10 284 (1.5)
Digoxin	1087 (1.5)	12 443 (1.8)
Tramadol	1077 (1.5)	13 107 (1.9)
Bumetanide	1066 (1.5)	6733 (1.0)
Fluoxetine	961 (1.3)	9163 (1.4)
Isosorbide	942 (1.3)	12 981 (1.9)
Fexofenadine	769 (1.1)	9350 (1.3)
Loperamide	749 (1.0)	11 299 (1.7)
Tolterodine	684 (1.0)	2687 (0.4)
Morphine	646 (0.9)	6854 (1.0)
Nifedipine	607 (0.8)	3132 (0.5)
Loratadine	590 (0.8)	12 195 (1.8)
Prochlorperazine	583 (0.8)	13 951 (2.1)
Buprenorphine	582 (0.8)	3480 (0.5)
Mebeverine	557 (0.8)	8971 (1.3)
Dihydrocodeine	529 (0.7)	3174 (0.5)
Metoprolol	462 (0.6)	1378 (0.2)
Betamethasone	430 (0.6)	16 094 (2.4)
Trospium chloride	423 (0.6)	1501 (0.2)
Colchicine	396 (0.6)	8273 (1.2)
Promethazine	373 (0.5)	109 (0.02)
Venlafaxine	336 (0.5)	3568 (0.5)
Carbamazepine	329 (0.5)	851 (0.1)
Diazepam	320 (0.4)	17 386 (2.6)
Risperidone	305 (0.4)	2778 (0.4)
Oxybutynin	300 (0.4)	4431 (0.7)
Trazodone	297 (0.4)	2069 (0.3)
Quetiapine	286 (0.4)	1951 (0.3)
Hyoscine	279 (0.4)	9385 (1.4)
Dosulepin	276 (0.4)	1208 (0.2)
Hydroxyzine	268 (0.4)	1080 (0.2)
Olanzapine	259 (0.4)	2208 (0.33)
Fentanyl	257 (0.4)	373 (0.06)
Nortriptyline	234 (0.3)	770 (0.1)
Paroxetine	215 (0.3)	3045 (0.45)
Chlorphenamine	209 (0.3)	3987 (0.6)
Ipratropium	209 (0.3)	1700 (0.3)
Hydrocortisone	203 (0.3)	28 614 (4.2)
Lorazepam	189 (0.3)	5971 (0.88)
Temazepam	173 (0.2)	4224 (0.62)
Oxycodone	172 (0.2)	1456 (0.21)
Alverine	149 (0.2)	1362 (0.2)
Escitalopram	148 (0.2)	734 (0.1)
Amisulpride	144 (0.2)	495 (0.07)
Glycopyrronium bromide	130 (0.2)	357 (0.05)
Desloratadine	126 (0.2)	271 (0.04)
Theophylline	124 (0.2)	70 (0.01)
Procyclidine	122 (0.2)	393 (0.06)
Baclofen	120 (0.2)	1637 (0.2)
Dipyridamole	117 (0.2)	1154 (0.17)
Clonazepam	114 (0.2)	1565 (0.2)
Cyclizine	110 (0.2)	12 735 (1.9)
Hydrochlorothiazide	110 (0.2)	874 (0.1)
Cinnarizine	108 (0.2)	2925 (0.4)
Haloperidol	95 (0.1)	1017 (0.2)
Flupentixol	92 (0.1)	327 (0.05)
Clonidine	90 (0.1)	550 (0.08)
Aripiprazole	87 (0.1)	1005 (0.15)
Fesoterodine	71 (0.1)	710 (0.1)
Captopril	57 (0.1)	366 (0.05)
Midazolam	57 (0.1)	5497 (0.81)
Clomipramine	55 (0.1)	410 (0.06)
Cimetidine	47 (0.1)	441 (0.07)
Atropine	42 (0.1)	294 (0.04)
Chlorpromazine	42 (0.1)	259 (0.04)
Imipramine	36 (0.1)	252 (0.04)
Darifenacin	30 (0.04)	1512 (0.2)
Nefopam	29 (0.04)	251 (0.04)
Trihexyphenidyl	27 (0.04)	184 (0.03)
Hydralazine	26 (0.04)	355 (0.05)
Amantadine	21 (0.03)	200 (0.03)
Indoramin	14 (0.02)	161 (0.02)

Medications prescribed to be taken as required were excluded in the development cohort but not in the external validation cohort, and therefore the prevalence of medications frequently prescribed as required (eg, hydrocortisone) was much higher in the external validation.

### Model development and internal validation

Table 3 shows the model coefficients. Under fivefold cross validation the model discrimination was good (bias corrected: C index=0.794 and Nagelkerke's R^2^=0.063) with a bias corrected calibration slope of 0.972. Based on month 1 anticholinergic drug use status, calibration was excellent when the predicted risk was <10% (accounting for 80% of patients), whereas we found some underestimation of risk for patients with a predicted risk of 10-25% (18% of patients), and some overestimation for patients with a predicted risk >25% (2% of patients) (figure 2). The baseline hazards at month 1 and month 12 were 0.3% and 2.9%, respectively.

**Table 3:** Model coefficients and anticholinergic medication index (ACMI) scores

Predictor	Coefficient	ACMI score
Age (−74)*	0.099	—
Age (−74)^2^^*^	−0.001	—
Sex (women *v* men)	−0.097	—
Electronic frailty index score (−0.0747)*	3.120	—
Memory or cognitive problems	0.557	—
Visual impairment	−0.045	—
Hearing impairment	−0.101	—
Parkinson’s disease	0.443	—
History of falls	0.493	—
Urinary incontinence	−0.002	—
Osteoporosis	−0.018	—
Arthritis	−0.174	—
Alverine	−0.760	0.5
Amantadine	1.806	2.3
Amisulpride	0.573	1.4
Amitriptyline	−0.071	1
Aripiprazole	0.625	1.5
Atenolol	−0.146	0.9
Atropine	1.177	1.9
Baclofen	0.335	1.3
Bendroflumethiazide	−0.483	0.7
Betamethasone	−0.609	0.6
Bumetanide	0.458	1.4
Buprenorphine	0.374	1.3
Captopril	−0.598	0.6
Carbamazepine	0.730	1.5
Cetirizine	−0.144	0.9
Chlorphenamine	0.198	1.2
Chlorpromazine	−0.446	0.7
Cimetidine	0.543	1.4
Cinnarizine	0.448	1.3
Citalopram	0.335	1.3
Clomipramine	−0.107	0.9
Clonazepam	0.166	1.1
Clonidine	−0.568	0.6
Codeine	0.334	1.3
Colchicine	−0.642	0.6
Cyclizine	0.897	1.7
Darifenacin	0.454	1.3
Desloratadine	−1.309	0.1
Dexamethasone	0.109	1.1
Diazepam	0.338	1.3
Digoxin	0.117	1.1
Dihydrocodeine	−0.170	0.9
Dipyridamole	−0.868	0.4
Dosulepin	−0.366	0.8
Doxazosin	−0.174	0.9
Escitalopram	0.735	1.6
Fentanyl	0.549	1.4
Fesoterodine	0.767	1.6
Fexofenadine	0.034	1
Fluoxetine	0.552	1.4
Flupentixol	0.086	1.1
Furosemide	0.180	1.1
Glycopyrronium bromide	−0.192	0.9
Haloperidol	0.604	1.5
Hydralazine	0.617	1.5
Hydrochlorothiazide	−0.266	0.8
Hydrocortisone	0.300	1.2
Hydroxyzine	−0.386	0.7
Hyoscine	0.293	1.2
Imipramine	−0.282	0.8
Indapamide	−0.447	0.7
Indoramin	0.591	1.4
Ipratropium	0.654	1.5
Isosorbide	0.044	1
Loperamide	−0.102	0.9
Loratadine	−1.070	0.2
Lorazepam	0.331	1.3
Mebeverine	−0.070	1
Metoprolol	0.223	1.2
Midazolam	1.081	1.8
Mirtazapine	0.367	1.3
Morphine	0.599	1.5
Nefopam	0.137	1.1
Nifedipine	0.161	1.1
Nortriptyline	−0.063	1
Olanzapine	0.661	1.5
Oxybutynin	0.231	1.2
Oxycodone	0.630	1.5
Paroxetine	0.213	1.2
Prednisolone	0.080	1.1
Prochlorperazine	−0.025	1
Procyclidine	0.665	1.5
Promethazine	−0.158	0.9
Quetiapine	0.378	1.3
Ranitidine	0.394	1.3
Risperidone	0.390	1.3
Solifenacin	−0.246	0.8
Temazepam	0.289	1.2
Theophylline	−0.015	1
Tiotropium bromide	0.301	1.2
Tolterodine	0.014	1
Tramadol	0.460	1.4
Trazodone	0.015	1
Trihexyphenidyl	0.919	1.7
Trospium chloride	0.385	1.3
Umeclidinium	0.270	1.2
Venlafaxine	0.409	1.3
Warfarin	−0.470	0.7

These coefficients should not be interpreted as distinct and unbiased quantifications of the relation with the dependent variable.

* Centred around the median (age=74, electronic frailty index score=0.0747).

**Figure 2:**
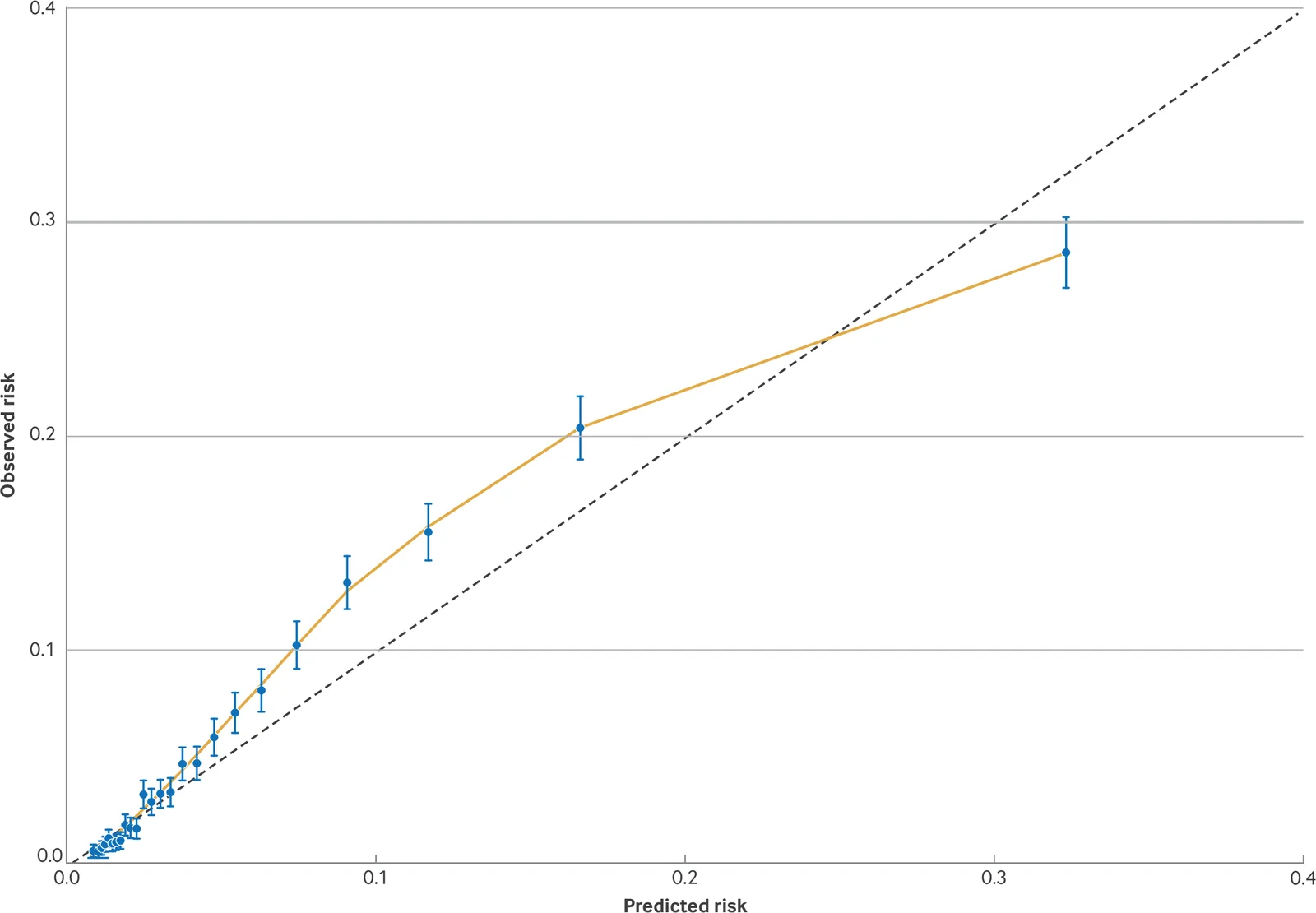
Development cohort calibration plot showing the predicted versus observed risk of admission to hospital for falls or delirium at month 12

### External validation

The model performed well in external validation, with good discrimination (C index=0.721, 95% CI 0.718 to 0.723, R^2^=0.040) but some degree of miscalibration (calibration slope=0.689) and some overall underestimation of risk (observed/expected=1.071). Based on month 1 anticholinergic drug use, the risk at month 12 was slightly underestimated when the predicted risk was <20% and overestimated for patients with a predicted risk >30% (2% of patients) (figure 3). Although the calibration slope was comparable across index of multiple deprivation groups, underestimation of risk was slightly increased in the most deprived group (figure 4).

**Figure 3:**
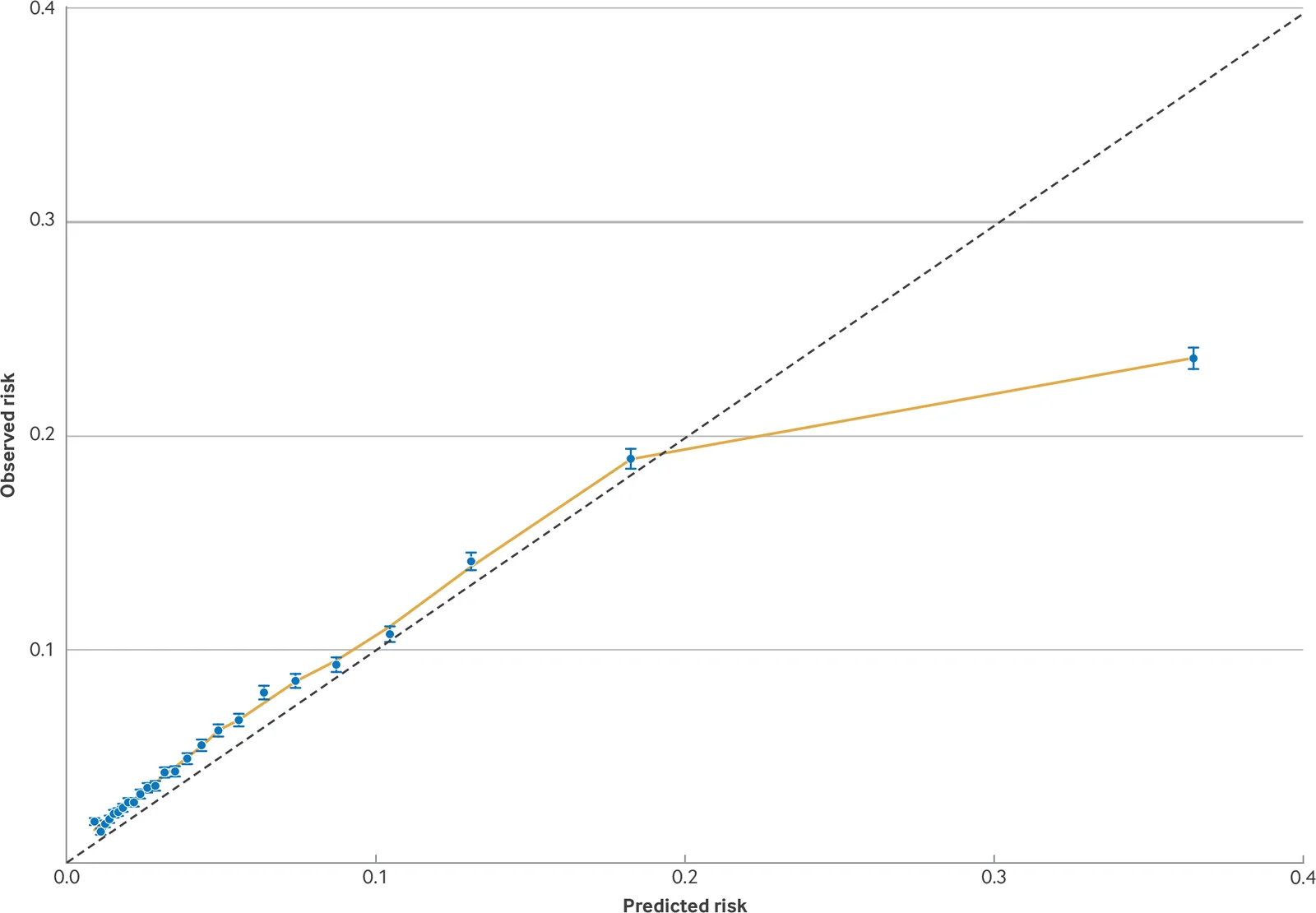
External validation calibration plot showing the predicted versus observed risk of admission to hospital for falls or delirium at month 12

**Figure 4:**
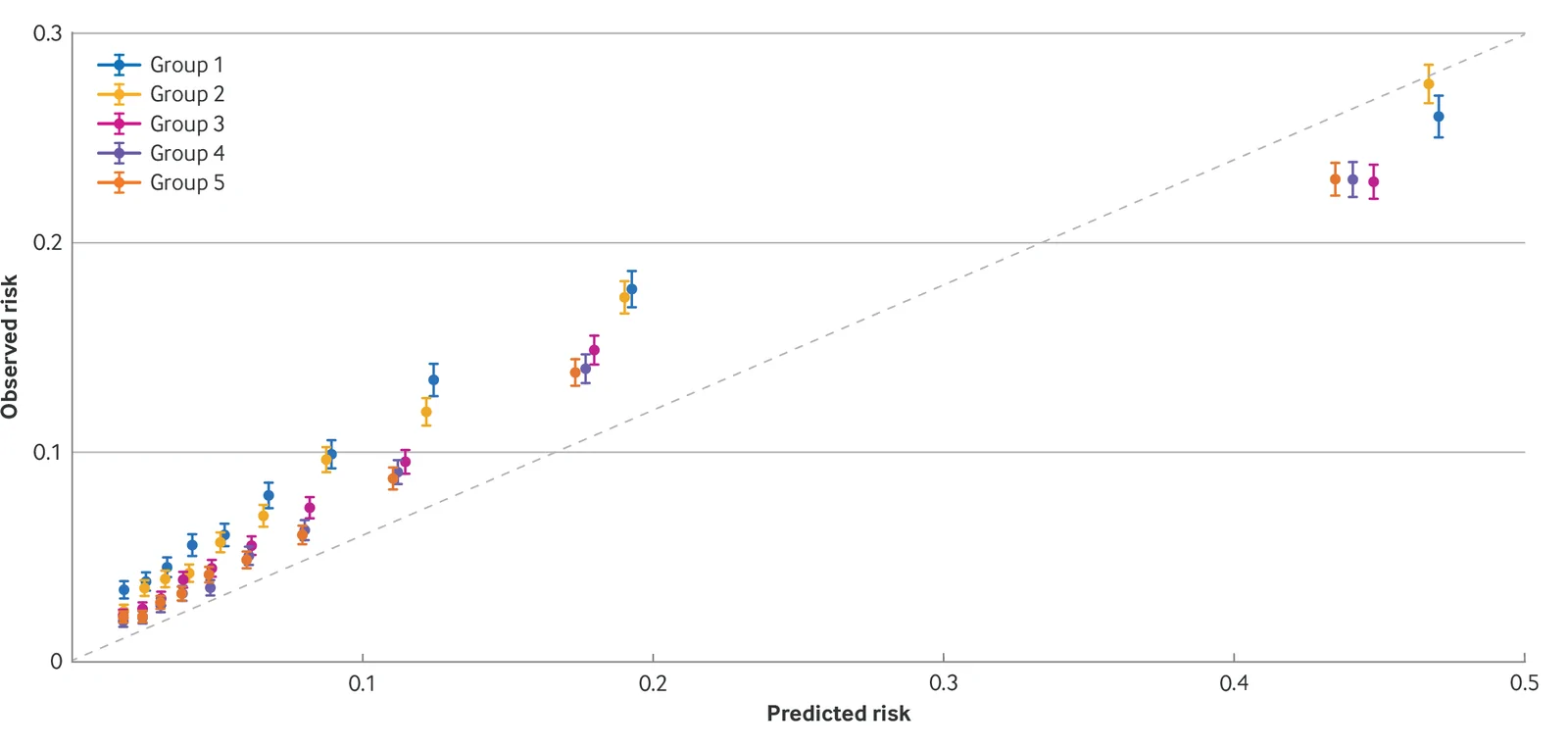
Calibration plot according to the Welsh index of multiple deprivation group in the external validation cohort. Index of multiple deprivation is categorised as five equal groups (groups 1-5), with group 1 being the most deprived and group 5 the least deprived group

### Derivation of anticholinergic medication index

The 88 anticholinergic medications were transformed (table 3), with the lowest score being 0.1 (desloratadine) and the highest 2.3 (amantadine). Online supplemental box 1 gives an example calculation of the predicted risk and ACMI score.

### Comparison with an existing scale

The clinical prediction model featuring the anticholinergic cognitive burden score had good discrimination in the development cohort (C index=0.781 and Nagelkerke's R^2^=0.059).

## Discussion

### Principal findings

The ACMI is directly linked to real world adverse events that disproportionately affect older or more vulnerable patients. We designed the ACMI in partnership with clinicians, patients, and electronic health record system suppliers to ensure its suitability for future use, enabling automated calculation of anticholinergic burden in patients aged >65 years. Used alongside predicted one year risk of delirium or falls, the ACMI can support clinicians in identifying and prioritising patients at high risk for structured medication reviews and potential deprescribing.

### Strengths and limitations of this study

The ACMI is strengthened by its derivation from a validated prediction model based on data from almost 750 000 UK patients aged >65 years. We performed internal and external validation of the model, in alignment with the Prognosis Research Strategy framework, to ensure methodological rigour,^44^ with good prognostic performance. The ACMI has been developed to be transferable to other settings, including internationally, with use of international non-proprietary drug names and mapping to internationally implemented coding systems supporting interoperability and deployment across different healthcare systems, after further validation. A recent external validation of ACMI for the risk of dementia with US data from the All of Us study provided supportive evidence for international transferability.^45^

We classed use of anticholinergic drugs as binary because incorporating dose has major deployment challenges, making it currently unfeasible for real time decision support. Although our alignment with deployment is a strength of the study and minimises the potential for research waste, one potential consequence is that the ACMI may be overestimated in patients on lower than average doses, and vice versa. Also, evidence exists indicating that the anticholinergic effect of some drugs has only been shown at high doses^46^ or in those with severe frailty.

We used time varying covariates to enable a patient's drug use status to change over the study period. This method was important because some of the most prescribed anticholinergic medications (eg, diuretics, opioids, and steroids) have a short half-life, meaning their pharmacological effects fluctuate quickly. This approach means, however, that the modelled risk of falls and delirium at one year would best be estimated with anticholinergic drug use at that time. In implementation, a patient's future anticholinergic use status will not be known, and therefore we performed our external validation with known drug use at month 1 as a proxy, to ensure that the implementation model was aligned with how it would be used in clinical practice.

We aggregated predictors into calendar months to reduce computational burden. Therefore, a patient prescribed a medication at any point during the month was classed as using the drug for the whole month. This method introduces potential misclassification of drug use when an outcome occurred before the prescription start date, which decreases the ACMI score. Because most medications were prescribed for a median duration of five months within the 12 month study period, however, the risk of misclassification was limited to a smaller number of prescriptions not yet established on repeat. Misclassification of drug use may also be a concern because of medication non-adherence.

Although the model was strengthened by the exclusion of medications prescribed to be taken as required from the development cohort, for the external validation cohort, exclusion of as required prescriptions was not possible because we did not have access to the prescription signature (free text prescription instructions). This approach contributed to the variation in prescribing rates for medications such as hydrocortisone and betamethasone that are commonly prescribed to be taken as required (0.3% *v* 4.2% and 0.6% *v* 2.4%). Consequently, anticholinergic burden may have been overestimated in a small proportion of patients (potentially 4% and 2%) in the external validation cohort and the predicted risk from the model slightly overinflated, proportional to the difference in prescribing rates.

In external validation, we found some overestimation of risk among high risk individuals, which may reflect overfitting in the development cohort, particularly given the relatively small number of patients with a very high predicted risk. The risk of falls or delirium may plateau beyond a certain threshold of anticholinergic drug use, resulting in a ceiling effect that the model does not account for. Individuals with advanced age, greater frailty, or multiple comorbidities may be prescribed anticholinergic medications at lower doses or with greater caution, resulting in lower actual drug use than the model assumes. These factors may contribute to the miscalibration in the upper range of predicted risk. In implementation, however, we anticipate that patients are selected for a structured medication review based on ranking of risk within a practice, and therefore the ACMI would still effectively prioritise patients.

Our approach to deriving the ACMI was predictive, which means that we cannot be certain of the causal effects of the included medications on falls and delirium. Therefore, we recommend using the overall ACMI score or predicted risk to identify or prioritise patients at high risk of anticholinergic side effects, but to use clinical judgment when assessing whether deprescribing an individual anticholinergic medication is clinically appropriate, rather than making judgments based on individual anticholinergic drug scores.

Whereas the ACMI scored 88 medications from 0.1 to 2.3 based on coefficients derived from a clinical prediction model, the anticholinergic cognitive burden scale scored 88 medications from one to three (in whole numbers) based on a scoring system devised by an interdisciplinary team in combination with published evidence.^29^ Only 44 medications appear in both scales because the anticholinergic cognitive burden scale is mainly US based and the ACMI was developed with UK data. In the anticholinergic cognitive burden scale, drugs scored as one are those with possible anticholinergic effects, and drugs scored as two or three are those with established clinically relevant cognitive anticholinergic effects.^29^ The distinction between anticholinergic medications scored as two or three in the anticholinergic cognitive burden scale is based on blood-brain barrier permeability and the association with the development of delirium. For the 44 medications in both scales, we found some consistency in scoring (online supplemental table 1). For example, most medications that scored one in the anticholinergic cognitive burden scale scored ≤1.5 in the ACMI. Seven anticholinergic medications, however, that scored three in the anticholinergic cognitive burden scale scored ≤1 in the ACMI (amitriptyline, chlorpromazine, hydroxyzine, imipramine, nortriptyline, promethazine, and tolterodine). Although neither the ACMI nor the anticholinergic cognitive burden scale incorporated dose, the lower ACMI weighting could represent more contemporary prescribing practices because some anticholinergic medications that have historically been prescribed in high doses (eg, tricyclic antidepressants) are now prescribed at much lower doses for alternative indications, typically the management of neuropathic pain.^47,48^

### Study implications

The ACMI is designed for integration into primary care electronic health record systems, enabling automated scoring for all patients aged >65 years within a practice. Current UK practice is to use the anticholinergic cognitive burden scale, either by manually adding medication scores or by entering medications into a webpage for individual patients.^49^ The ACMI offers an efficient, scalable solution to reduce clinician burden and support targeting of patients who may benefit most from a structured medication review. Our work in partnership with suppliers of the main UK primary care electronic health record systems to ensure that the developed risk prediction model and ACMI are suitable for routine use closely aligns with UK policy and practice priorities (eg, the NHS England Primary Care Network contract specifies targeted structured medication reviews for people at high risk of adverse outcomes).^50^ To support future use, we have piloted implementation of the ACMI into electronic health records in one primary care network to automatically derive the list of anticholinergic medications currently prescribed, the ACMI score, and the one year predicted risk of falls or delirium for each patient aged >65 years in a practice. The practice can rank patients according to their ACMI score or predicted risk, or both, and invite those with the highest scores for a structured medication review. Online supplemental box 2 shows examples of the ACMI output that clinicians can use to rank the ACMI score and predicted risk (one year predicted risk of a hospital admission for a fall, fracture, or delirium (composite outcome)).

Future research should align with the evidence hierarchy for clinical prediction models,^51^ including further external validation to examine the performance of the ACMI in international primary care settings and in secondary care. Model impact assessment should evaluate performance in clinical settings, including co-development of integrated clinical decision support systems and robust practitioner training packages that look at knowledge gaps, patient concerns about deprescribing, and other barriers such as clinician autonomy because of organisational arrangements. These challenges are highly relevant for anticholinergic medications and must be considered as part of any future implementation and evaluation plans. International guidelines on deprescribing anticholinergic medications have been developed and should be considered as part of robust practitioner training.^52^ Our extensive work to align the ACMI with future UK and international deployment will maximise the potential for clinical impact, targeting a potentially modifiable but often overlooked contributor to adverse outcomes of major importance to patients and healthcare systems in the UK and internationally.^52^

## Supplementary material

10.1136/bmjmed-2026-002835 online supplemental file 1

10.1136/bmjmed-2026-002835 online supplemental file 2

10.1136/bmjmed-2026-002835 online supplemental file 3

10.1136/bmjmed-2026-002835 online supplemental table 1

## Data Availability

Data may be obtained from a third party and are not publicly available.
