# Supplementary material for: Deriving the anticholinergic medication index for delirium and falls in older people: development and external validation of a risk prediction model
Source: BMJ Med. 2026 Nov 4;5(1):e002835. doi: 10.1136/bmjmed-2026-002835 (PMC13735312; doi:10.1136/bmjmed-2026-002835)
Supplement: Supplementary file 1 [file bmjmed-2026-002835.reviewer_comments.pdf]

PEER REVIEW HISTORY

BMJ Medicine publishes all reviews undertaken for accepted manuscripts. Reviewers are asked to complete a checklist review form and are provided with free text boxes to elaborate on their assessment. These free text comments are reproduced below.

This paper was submitted to another journal from BMJ but declined for publication following peer review. The authors addressed the reviewers' comments and submitted the revised paper to BMJ Medicine. The paper was subsequently accepted for publication at BMJ Medicine.

ARTICLE DETAILS

**TITLE (PROVISIONAL)**	Deriving the Anticholinergic Medication Index (ACMI): development and external validation of a risk prediction model for delirium and falls in older people
**AUTHORS**	Best, Kate; Butters, Olly; Schofield, Pieta Georgina; Todd, Oliver; Alderson, Sarah; Alldred, David; Bonnett, Laura; Buchan, Iain; Farrin, Amanda; Johnson, Owen; McInerney, Ciarán; Mehdizadeh, David; Lawton, Thomas; Lawton, Rebecca; Shuweidhi, Farag; Walker, Lauren E; West, Robert; Young, Bridget; Pirmohamed, Munir; Copsey, Bethan; Abuzour, Aseel; Rodgers, Sarah; Clegg, Andrew

VERSION 1 - REVIEW

**REVIEWER NAME**	Reviewer 1
**REVIEWER AFFILIATION**	
**REVIEWER CONFLICT OF INTEREST**	None
**DATE REVIEW RETURNED**	19-Sep-2025

**GENERAL COMMENTS**	1. Are the questions the paper addresses relevant and important to patients and/or carers?<br>- The study investigates how medications with anticholinergic effects (which are commonly prescribed to older adults) can increase the risk of falls and delirium (which are potentially life-threatening). Anything that can help reduce these risks (e.g., a tool to assess medication burden) is relevant and beneficial to patients and carers.<br><br>2. Are there topics or issues that are missing, or need to be highlighted more?<br>- It would be helpful if the authors included a visual summary of (1) the development and validation process of the tool; and (2) how ACMI would look and work in a real clinical setting.<br><br>3. Is the treatment or intervention suggested or guidance given something which patients/carers can readily take up? or does it present challenges?<br>- The intervention (a scoring system built into electronic records that flags high anticholinergic burden) seems practical. However, there may be challenges where patients feel anxious about changing long -standing prescriptions, especially if they perceive them as beneficial.<br>- Are there any non-pharmaceutical alternatives that could be considered when reducing anticholinergic medications? What does

	the literature look like? Would that be helpful when discussing medication reductions with patients?<br><br>4. Are the outcomes described/measured in the study important to patients/carers? Are there others that should have been considered?<br>- Falls and delirium are hugely important outcomes to patients and carers. Would it be possible to explore other possible outcomes of interest to patients, such as cognitive decline and reduced quality of life?<br><br>5. Do you have any suggestions that might help the author(s) strengthen their paper and make it more useful for doctors to share and discuss with patients/ carers?<br>- "For the development cohort, Bradford and Craven residents aged >65 years on the 1st January 2019 who contributed data to Connected Bradford between 1st January 2019 and 31st December 2019 were used." Why only data within 2019 was used? Would that cause selection bias and skew the results? Authors mentioned "some anticholinergic medications that have historically been prescribed in high dose (for example tricyclic antidepressants) are now prescribed at much lower dose" – would the restriction in data affect the interpretation of results?<br>- It would be preferrable to include a patient-friendly explanation or summary of the ACMI, which the PPI group may be helping with. Given there are a number of considerations to take into account of when interpreting the ACMI score (e.g., "there are 7 anticholinergic medications scoring 3 in the ACB scale that score 1 or less in the ACMI", it would be beneficial to provide some simple guidelines on how to discuss medication reductions.<br><br>6. Do you think the level of patient/carer involvement in the study could have been improved? If there was none do you have ideas on how they might have done so?<br>- The authors established a PPI group consisting of 7 lay representatives with lived experience of taking multiple medications, which is great. This group helped steered the project and identified outcomes of importance. To improve further, it would be helpful if: (1) the authors had quoted or described specific feedback or concerns raised by the PPI group; and (2) future work includes testing the tool's acceptability with patients or carers (i.e., not just with clinicians).

**REVIEWER NAME**	Reviewer 2
**REVIEWER AFFILIATION**	
**REVIEWER CONFLICT OF INTEREST**	None
**DATE REVIEW RETURNED**	19-Sep-2025

**GENERAL COMMENTS**	The study aimed to derive a new measure of anticholinergic burden, the Anticholinergic Medication Index (ACMI), by converting the weights of each medication into coefficients within a clinical prediction model for falls and delirium. The model incorporates anticholinergic medications as predictors. This is an innovative and well-designed study conducted on a large sample of older adults and addresses an important clinical issue: the adverse effects of anticholinergic medications in this population. Notably, it is the first study to validate the anticholinergic potency of individual

	medications based on clinically relevant outcomes—falls and delirium—which could have meaningful implications for practice.<br>The references cited are appropriate and up-to-date.<br>I have the following comments for the authors:<br>1. Could the authors provide a list of medications included in the ACMI but not present in the ACB scale? This would enhance comparisons between the two tools.<br>2. The study compared the ACMI with the ACB scale, and a clinical prediction model incorporating the ACB score was also developed. However, the potential advantages of the ACMI over the ACB scale could be more clearly discussed.<br>3. Did the authors consider the possibility of suggesting a cut-off value for the ACMI to identify patients with a high anticholinergic burden?<br>4. I partially disagree with the statement: "However, the requirement for the ACB score to be calculated manually by clinicians means it has been difficult to translate this tool into routine care." In fact, an online ACB calculator is available (https://www.acbcalc.com/), and some decision support systems (e.g., INTERCheck in Italy: Ghibelli S et al., Drugs Aging. 2013;30(10):821-8. doi:10.1007/s40266-013-0109-5) automatically calculate the anticholinergic burden according to the ACB scale. Therefore, I would argue that integrating ACMI into electronic health records may not be easier than implementing the ACB scale.<br>5. If medication adherence was not assessed in the study, this should be added in the Limitations section

**REVIEWER NAME**	Reviewer 3
**REVIEWER AFFILIATION**	
**REVIEWER CONFLICT OF INTEREST**	None
**DATE REVIEW RETURNED**	19-Sep-2025

**GENERAL COMMENTS**	Abstract:<br><br>Line 14. Range of conditions including incontinence ?? presumably urinary. OAB more appropriate…<br><br>Background:<br>Page 8 Line 16. Spasmolytics for urinary incontinence. Perhaps antimuscarinics for OAB is more accurate<br><br>Page 8 Line 46. Agree anticholinergic burden is modifiable and often overlooked, but the trial evidence dose not support a reduction in burden improves outcomes. I suggest, this clinical equipoise must be made clear upfront.<br><br>Page 10 Lines 26,27. Why were falls and delirium chosen as outcomes to validate the model?<br>Why did the authors choose a composite model? Did the authors validate the model performance for falls and delirium as separate outcomes?<br>Approximately 1or 2 out of 100 community patients may be diagnosed with delirium? Do we have any epidemiological studies to show the prevalence of ACh burden in patients with delirium?<br>Since outcomes were sourced from secondary care data & notoriously delirium is poorly coded- does this does not introduce misclassification?

	Page 11 Line 46. Suggest providing more clarity on how drug exposure was calculated. Would a prescribed dose of amitriptyline 25 mg and 75 mg be assigned the same score?<br><br>Page 12 Line 51. Drug exposure was coded as binary… is this a massive limitation?<br><br>Figure 2: the calibration plot underpredicts at 0.2-0.4? could the authors please comment on this?<br><br>Discussion:<br>Page 20, Line 13, 'Where existing anticholinergic burden tools are based on<br>theoretical receptor affinity or subjective scoring systems19 21, the ACMI offers a more<br>clinically relevant measure of anticholinergic burden'…<br>This has to rephrased. The ACMI index was predominantly derived (44/88) from the 88-medication listed in the original ACB scale. What can be potentially argued is the scalability of deriving an index such as ACMI that has been clinically validated for composite outcomes (delirium and falls) and like the electronic frailty index can be incorporated in EHR-this makes it useful for practitioners.<br><br>Page 21, Line 6. Future research should explore how incorporation of drug dose may improve prognostic performance.<br>Why not now? With huge advancements in data science and AI is this not feasible? The incorporation of dose into exposure calculation will make this index more accurate and would be an innovatyion.<br><br>Page 22, line 26. I agree with the authors ACMI can be used to trigger to initiate a medication review.<br><br>Arguably, there are 2 aspects to the discussion that needs to be covered.<br>a) Research utility of ACMI- since the ACMI scores are derived from coefficients of a composite model (e.g., weighted based on effect sizes for outcomes in your derivation cohort), and since anticholinergic burden is mechanistically linked to decreased mobility, cognitive decline, and impaired global function, can the ACMI be used as a proxy or predictor for these outcomes in new populations or further validation is required or is this a limitation of the ACMI.<br>b) Clinical utility- used as a trigger tool for a SMR when a patient experience falls and delirium.<br><br>Page 23, Lines 13 to 28 However, there are 7<br>….anticholinergic medications scoring 3 in the ACB scale that score 1 or less in the ACMI<br>(Amitriptyline, Chlorpromazine, Hydroxyzine, Imipramine, Nortriptyline, Promethazine,<br>Tolterodine). This could represent an important contemporary reflection of the ACMI, as<br>some anticholinergic medications that have historically been prescribed in high dose (for<br>example tricyclic antidepressants) are now prescribed at much lower dose for alternative<br>indications, typically management of neuropathic pain….

	The above paragraph should be qualified with appropriate references as it can be deemed to be speculative as dose was never a consideration when the ACB scale was derived nor the ACMI.<br><br>Clinical practitioners (e.g. pharmacists, general practitioners, advanced nurse<br>practitioners) could implement the tool in primary care to automatically derive the ACMI<br>score based on their current prescriptions, along with their one year predicted risk of<br>adverse outcomes, based on their age, sex, comorbidities and eFI score in addition to<br>current prescriptions<br><br>It seems that the model has not yet been validated for other global clinical outcomes. Could the others please clarify how ACMI might be applied for 'adverse outcomes,' as this connection is not immediately clear to me?<br><br>Table 3: Model coefficients and ACMI score<br>…interpreting these coefficients as distinct and unbiased quantifications<br>of the relationship with the dependent variable.<br><br>I agree with the potential benefits discussed; however, I believe we should remain cautious regarding the scalability of ACMI, especially in light of the limitations I've highlighted previously. Importantly, the coefficients and ACMI score will change based on the dependent variable?<br><br>Example<br>A 70-year-old female patient with an eFI score of 0.25 (moderate frailty), with arthritis, for which she is prescribed codeine, arthritis treated<br>with long-term, low-dose maintenance prednisolone and atrial fibrillation for which she is anticoagulated with warfarin.<br>LP= 0.099 x (70-74) -0.001 x (70-74)2 + 3 x (0.25-0.0747) -0.097 -0.174 +<br>0.3344 + 0.080 -0.470 = -0.2127<br>One year risk = 2.9% x exp(-0.2127) = 2.3%<br><br>Thank you to the authors for providing this example. I think the authors should provide a stronger clinical context here to help interpret the findings. If the 2.3% figure they report is the adjusted annual risk, calculated from the baseline 2.9% using a hazard ratio of roughly 0.81 (since exp(-0.2127). Is it possible to derive NNT to prevent one hospital admission following a deprescribing intervention (SMR)-I am assuming such an intervention is low-cost and feasible. I could be entirely wrong in my argument but would welcome any clarification.

**REVIEWER NAME**	Reviewer 4
**REVIEWER AFFILIATION**	
**REVIEWER CONFLICT OF INTEREST**	None
**DATE REVIEW RETURNED**	

GENERAL COMMENTS	The manuscript entitled: " Deriving the Anticholinergic Medication Index (ACMI): development and external validation of a risk prediction model for delirium and falls in older people" studied the development and validation of an index (ACMI) to predict the risk of delirium and falls in older adults from exposure to anticholinergic medications.<br><br>In my opinion, the study is interesting and its justification is adequate. A time-dependent Cox model was used, which allows for exposure to each medication change over time (month to month). This seems appropriate because many anticholinergic drugs are prescribed and discontinued throughout the year.<br><br>The model was adjusted for relevant clinical factors (age, sex, dementia, frailty, history of falls, etc.), making it clinically robust. Moreover, internal validation (cross-validation) and external validation (SAIL cohort, Wales) were performed, strengthening the methodological robustness.<br><br>However, the model has some limitations that are described by the authors:<br>- The model predicted the direction of risk well (C-index ~0.72), but the calibration was imperfect (calibration slope 0.69), meaning it tended to underestimate or overestimate in some groups.<br>- Only "exposed/unexposed" was considered by month, without including dose or cumulative duration. This simplifies implementation in EHRs, but may reduce accuracy, given that anticholinergic toxicity is often dose-dependent.<br>- PRN ("on-demand" medication) were excluded in the development cohort, but not in the validation cohort, which may have introduced heterogeneity and overestimated risk in the validation cohort.<br><br>On the other hand, a composite outcome (delirium + falls) was used, which may mask differences in predictive ability for each event separately (e.g., the model predicted delirium better in Bradford and falls better in Wales). Respect this, could the authors explain why a composite outcome has been used?

VERSION 1 – AUTHOR RESPONSE

**Reviewer 1**		
1. Are the questions the paper addresses relevant and important to patients and/or carers?<br>- The study investigates how medications with anticholinergic effects (which are commonly prescribed to older adults) can increase the risk of falls and delirium (which are potentially life-threatening). Anything that can help reduce these risks (e.g., a tool to assess medication burden) is relevant and beneficial to patients and carers.	Thank you for this comment	

2. Are there topics or issues that are missing, or need to be highlighted more?<br>- It would be helpful if the authors included a visual summary of (1) the development and validation process of the tool; and (2) how ACMI would look and work in a real clinical setting.	Thank you for this suggestion, we have added a flowchart to the supplementary materials as a visual summary of the development and validation process and cross referenced it in the methods.<br><br>We agree that it is important to describe what the ACMI would look like in a real clinical setting. In Supplementary Box 1, we had already included an example calculation of a patient's ACMI score and one year predicted risk. However, we have now added Supplementary Box 2, to show what the output that a clinician might see in practice, adding this footnote:<br><br>*In practice we recommend that the example ACMI output would be generated for all patients aged >65 in a practice and the patients are ordered by their ACMI score or their predicted risk, to help practices prioritise patients to invite for SMR.*<br><br>We have also added to the discussion:<br><br>*"The tool could be implemented in primary care software systems to automatically derive the list of AC medications prescribed currently, ACMI score and the one year predicted risk of fall or delirium, for each patient aged >65 in a practice. The practice can rank patients according to their ACMI score and/or predicted risk and invite those with the highest scores for structured medication reviews. Examples of the ACMI output that can be used by clinicians to rank ACMI score and predicted risk are shown in Supplementary Box 2."*<br><br>Please also note that we have conducted a pilot evaluation to assess the feasibility of implementation in general practices using the SystmOne electronic health record system in Bradford. We are preparing two additional manuscripts – one detailing the pilot evaluation, and another detailing how	Supplementary Figure 1<br><br>Supplementary Box 1<br><br>Discussion >pg27

	ACMI was translated from a model into a prototype clinical decision support system.	
3. Is the treatment or intervention suggested or guidance given something which patients/carers can readily take up? or does it present challenges?<br>- The intervention (a scoring system built into electronic records that flags high anticholinergic burden) seems practical. However, there may be challenges where patients feel anxious about changing long-standing prescriptions, especially if they perceive them as beneficial.<br>- Are there any non-pharmaceutical alternatives that could be considered when reducing anticholinergic medications? What does the literature look like? Would that be helpful when discussing medication reductions with patients?	We agree with the reviewer that these are important considerations. Structured Medication Reviews are recommended by the National Institute for Health and Care Excellence and as part of NHS England policy, with anticholinergic medications identified as a potential target. A key challenge has been selection of patients at high risk of anticholinergic medication-related side effects, which formed the rationale for this work.<br><br>Following a pilot implementation of the ACMI in Bradford (that included a training package to help address the relevant issues raised by the reviewer) primary care pharmacists were interviewed. They reported that patients experiencing discomfort from side effects were more accepting and willing to change their medicines to reduce their overall risk of falls/delirium. Challenges around deprescribing included the lack of autonomy in changing medicines due to organisational agreements, and the need to collaborate with doctors such as psychiatrists for medicines such as antipsychotics which primary care pharmacists typically would not alter themselves without the input of the main prescriber. Primary care pharmacists did report on patients' reluctance to changes in their medicines – this included opioids and some antidepressants.<br><br>These findings highlight the importance of integrating the ACMI with relevant organisational considerations as part of a wider model of care for deprescribing. This will be set out in detail in our planned paper on the feasibility of implementation of the ACMI, so we have not included this in the present paper (also due to word count constraints). We have added to the discussion: *"There are well-recognised challenges of implementing Structured Medicication Reviews, including practitioner training, concerns about stopping long-*	

	standing prescriptions, and lack of clinician automony due to organisational arrangements. These challenges are highly relevant for anticholinergic medications and must be considered as part of any future implementation and evaluation plans. Guidelines have been developed for deprescribing anticholinergic mediciations and can be considered to support future implementation."<br><br>We agree that clinicians could discuss non-pharmacological strategies where deprescribing of AC medications is recommended. However, the type of non-pharmacological intervention will necessarily vary according to the underlying indication for the AC medication, and can therefore encompass many different types of interventions (e.g. physical therapy/ exercise/ acupuncture for pain, CBT for insomnia, bladder training/ pelvic floor exercises for urinary incontinence). We therefore feel that this is outside the scope of our paper, and since guidelines for clinicians involved in deprescribing are available elsewhere, we have not covered this in detail in our paper, instead signposted to them as above.	Discussion > pg 27
4. Are the outcomes described/measured in the study important to patients/carers? Are there others that should have been considered?<br>- Falls and delirium are hugely important outcomes to patients and carers. Would it be possible to explore other possible outcomes of interest to patients, such as cognitive decline and reduced quality of life?	As outlined in our earlier response, falls and delirium are of major importance to the patients and public members that we consulted at the beginning and throughout the study, and there is a strong evidence base on the association between anticholinergic burden and these outcomes. Falls and delirium have major collective impact on the health and care system, including considerable economic impact.<br><br>We do agree with the reviewer that other outcomes are also important. As outlined in our earlier response, we have recently completed and published an external validation of the ACMI in UK Biobank and the US All of Us study, identifying that a higher ACMI score indicates a higher risk of cognitive decline{Asiimwe, 2025 #55}.	

	Additionally, we are leading follow-on HDR UK-funded work to investigate validity of the ACMI score in secondary care, with linked NIHR-funded work to investigate development of a Clinical Decision Support System integrated with the Oracle/Cerner EPR system.	
5. Do you have any suggestions that might help the author(s) strengthen their paper and make it more useful for doctors to share and discuss with patients/ carers?<br><br>- "For the development cohort, Bradford and Craven residents aged >65 years on the 1st January 2019 who contributed data to Connected Bradford between 1st January 2019 and 31st December 2019 were used." Why only data within 2019 was used? Would that cause selection bias and skew the results? Authors mentioned "some anticholinergic medications that have historically been prescribed in high dose (for example tricyclic antidepressants) are now prescribed at much lower dose" – would the restriction in data affect the interpretation of results?<br><br>- It would be preferrable to include a patient-friendly explanation or summary of the ACMI, which the PPI group may be helping with. Given there are a number of considerations to take into account of when interpreting the ACMI score (e.g., "there are 7 anticholinergic medications scoring 3 in the ACB scale that score 1 or less in the ACMI", it would be beneficial to provide some simple guidelines on how to discuss medication reductions.	Our a priori sample size calculations indicated that one year of data would provide sufficient patients and outcomes to robustly estimate a risk prediction model. At the time of analysis, this was the most recent pre-pandemic year available to the research team, making it the most appropriate dataset for model development. Our reference to historically higher doses being prescribed considerably pre-dates 2019.<br><br>We agree that a simple guideline on how to explain the ACMI score to patients would be most beneficial. The co-produced training package that we developed in collaboration with a clinical reference group and PPIE group (as part of the wider ACMI project) includes considerations when discussing 'risk' with patients. This included framing health messages to discuss risk with patients. For example, a gain-framed appeal would include emphasising potential benefits from stopping medications, whilst a loss-framed approach would include preventing future loss of function (e.g. cognitive function).<br><br>PPIE stated that any framing of risk, whether in the context of a gain-framed or loss-framed appeal had to be on a case by case basis with the patient. The PPIE group also included a case example in the training package as a way for pharmacists to consider how to approach the patient/carer to reduce/switch/stop AC medicines.	
6. Do you think the level of patient/carer involvement in the study could have been improved? If there was none do you have	We have added to the methods> PPI section: "*The PPI group gave input into what the ACMI score should look like, how it*	Methods> PPI

ideas on how they might have done so?<br>- The authors established a PPI group consisting of 7 lay representatives with lived experience of taking multiple medications, which is great. This group helped steered the project and identified outcomes of importance. To improve further, it would be helpful if: (1) the authors had quoted or described specific feedback or concerns raised by the PPI group; and (2) future work includes testing the tool's acceptability with patients or carers (i.e., not just with clinicians).	*might be interpreted by patients and how risk should be communicated to patients."*<br><br>As part of the wider project, the PPIE group was heavily involved in the co-production of training resources that would be used by the primary care pharmacists that would test the feasibility of implementing the ACMI into their practice during the process of conducting SMRs.<br><br>More specific examples of PPI feedback included the need for patients to understand potential side effects they may be experiencing as patients do not always recognise the side effects as 'side effects' after a long period of being on a certain medication. The PPIE group was concerned that inviting patients for an SMR using the ACMI tool would be to reduce costs to the NHS rather than reduce the risk of potential harm to patients. The PPIE group also noted that any discussion of risk to patients should include that it is 'potential' risk and that the method of communicating risk should be integrated into individualised shared decision making. We have not included these examples in the present paper but they will be incorporated in further publications on the pilot evaluation of the ACMI in a clinical practice in Bradford<br><br>We agree with the reviewer that future work must include testing acceptability with patients/carers, including their understanding of ACMI and how this may impact on their health. We have added to the discussion "*Evaluation of the ACMI in clinical practice is also recommended to test feasibility and determine both clinician and patient acceptability.* "	

		Discussion<br>> pg 27
**Reviewer 2**		
1. Could the authors provide a list of medications included in the ACMI but not present in the ACB scale? This would enhance comparisons between the two tools.	Thank you for this comment, we have added a supplementary table to show the ACB and ACMI scores of individual drugs in the ACB. We have referred to this table in the Discussion:<br><br>*"For the 43 medications present in both scales, there is some consistency in scoring (see Supplementary Table 1)."*	Supplementary table 1 and discussion
2. The study compared the ACMI with the ACB scale, and a clinical prediction model incorporating the ACB score was also developed. However, the potential advantages of the ACMI over the ACB scale could be more clearly discussed.	Thank you, we have added to the discussion:<br><br>*"The ACMI is designed for integration into primary care software systems, enabling automated score derivation for all patients aged >65 within a practice. Current UK practice in the UK is to use the ACB scale, either by manually adding medication scores or by entering medications into a webpage, for individual patients {Rebecca King, 2024 #52}. The ACMI offers an efficient, scalable solution to reduce burden on clinicians and support targeting of patients who may benefit most from a structured medication review."*	Discussion<br><br>>Pg 26
3. Did the authors consider the possibility of suggesting a cut-off value for the ACMI to	Please see our response to the Stats Editor-comment 5	

identify patients with a high anticholinergic burden?		
4. I partially disagree with the statement: "However, the requirement for the ACB score to be calculated manually by clinicians means it has been difficult to translate this tool into routine care." In fact, an online ACB calculator is available (https://www.acbcalc.com/), and some decision support systems (e.g., INTERCheck in Italy: Ghibelli S et al., Drugs Aging. 2013;30(10):821-8. doi:10.1007/s40266-013-0109-5) automatically calculate the anticholinergic burden according to the ACB scale. Therefore, I would argue that integrating ACMI into electronic health records may not be easier than implementing the ACB scale.	The ACB calculator and INTERcheck require manual entry of patient information or extraction of data into a different software, respectively. The ACMI has been designed to be integrated directly into primary care software systems, which makes it less burdensome and more time efficient. However, we have now mentioned the online calculator within the background section:<br><br>"*However, the requirement for the ACB score to be calculated manually or via data input into a website (https://www.acbcalc.com), by clinicians means it has been difficult to translate this tool into routine care.*"<br><br>In the discussion we have also added:<br><br>*The ACMI is designed for integration into primary care software systems, enabling automated score derivation for all patients aged >65 within a practice. Current UK practice in the UK is to use the ACB scale, either by manually adding medication scores or by entering medications into a webpage, for individual patients {Rebecca King, 2024 #52}. The ACMI offers an efficient, scalable solution to reduce burden on clinicians and support targeting of patients who may benefit most from a structured medication review.*	Background> 8<br><br>Discussion >pg 27
5. If medication adherence was not assessed in the study, this should be added in the Limitations section	We have added to the discussion: *"Exposure misclassification may also be an issue due to medication adherence.*"	Discussion >pg24
**Reviewer 3**		
Abstract:<br><br>Line 14. Range of conditions including incontinence ?? presumably urinary. OAB more appropriate…	Thank you we have changes incontinence to overactive bladder.	Abstract

Background:<br>Page 8 Line 16. Spasmolytics for urinary incontinence. Perhaps antimuscarinics for OAB is more accurate	We have altered to *"antimuscarinics for overactive bladder and intestinal spasm"*	Background>
Page 8 Line 46. Agree anticholinergic burden is modifiable and often overlooked, but the trial evidence dose not support a reduction in burden improves outcomes. I suggest, this clinical equipoise must be made clear upfront.	We have added the following:<br>*"Anticholinergic burden is modifiable and often overlooked, but there is still uncertainty from available trial evidence regarding reductions in AC burden from deprescribing interventions and improvement in clinical outcomes (Braithwaite et al 2023)"*	Background> g 7
Page 10 Lines 26,27. Why were falls and delirium chosen as outcomes to validate the model?<br>Why did the authors choose a composite model? Did the authors validate the model performance for falls and delirium as separate outcomes?<br>Approximately 1or 2 out of 100 community patients may be diagnosed with delirium?<br>Do we have any epidemiological studies to show the prevalence of ACh burden in patients with delirium?<br>Since outcomes were sourced from secondary care data & notoriously delirium is poorly coded- does this does not introduce misclassification?	Please see our answer to 'Editors comments #3', in relation to composite outcome.<br><br>We agree that coding of delirium is imperfect in secondary care, although there is evidence that this has improved during 2012 -2020 {Ibitoye, 2023 #64}. We<br><br>anticipate the misclassification of delirium is more likely to be false negative than false positive, which may mean we have some underestimation of risk.However, there is no evidence to suggest that under-reporting would vary according to anticholinergic exposure, which suggests our AC coefficient should be robust.	
Page 11 Line 46. Suggest providing more clarity on how drug exposure was calculated. Would a prescribed dose of amitriptyline 25 mg and 75 mg be assigned the same score?	Yes, a prescribed dose of 25mg or 75mg would both be treated the same, as exposed. As stated in the methods *"Exposure to each medication was coded as binary (exposed/ unexposed in each month). Following discussions with suppliers of primary care EHR systems, all forms of medication (e.g. oral solution/ tablet) were combined, and drug dose was not represented in the model, because it is unfeasible to accurately estimate drug dose within EHR systems, with negative impact on model deployment."*<br><br>We have further elaborated on the	Methods> predictors> pg 14

	pragmatic decision to treat anticholinergic exposure as binary, due to implementation constraints, in our response to 'Other Editors comment #1'	
Page 12 Line 51. Drug exposure was coded as binary… is this a massive limitation?	Coding drug dose as binary was a necessary limitation to enable integration within primary care software systems. Attempts to include drug dose would have led to a model that would be unfeasible to deploy, risking research waste. Please see our response to 'Other Editors comment #1', for more detail	
Figure 2: the calibration plot underpredicts at 0.2-0.4? could the authors please comment on this?	Thank you for highlighting the mis-calibration. For those with predicted risk >0.2, the model overpredicts their risk (in the external validation). In line with Stats editor comment #4, we addressed this in the Discussion *"In the external validation there was some overestimation of risk…"*	Discussion > pg 24-25
Discussion:<br>Page 20, Line 13, 'Where existing anticholinergic burden tools are based on theoretical receptor affinity or subjective scoring systems19 21, the ACMI offers a more<br>clinically relevant measure of anticholinergic burden'…<br>This has to rephrased. The ACMI index was predominantly derived (44/88) from the 88-medication listed in the original ACB scale. What can be potentially argued is the scalability of deriving an index such as ACMI that has been clinically validated for composite outcomes (delirium and falls) and like the electronic frailty index can be incorporated in EHR-this makes it useful for practitioners.	Thank you, our intention here was to comment on the way ACMI (and ACB) score the AC medications, rather than on the choice of medications included in the scale. However, we have removed this sentence to focus more on the scalability of the ACMI.	Discussion paragraph 1
Page 21, Line 6. Future research should explore how incorporation of drug dose may improve prognostic performance.<br>Why not now? With huge advancements in data science and AI is this not feasible? The incorporation of dose into exposure calculation will make this index more accurate and would be an innovation.	Whilst we agree that with advances in data science approaches it may be possible to incorporate drug dose into a prediction model, there would be major challenges in implement the resulting model into existing primary care software systems, risking research waste through development of a model that is unfeasible to deploy. For	

	further information, please see our response to 'Other Editors, comment #1'	
Page 22, line 26. I agree with the authors ACMI can be used to trigger to initiate a medication review.	Thank you for this comment	
Arguably, there are 2 aspects to the discussion that needs to be covered.<br>a) Research utility of ACMI- since the ACMI scores are derived from coefficients of a composite model (e.g., weighted based on effect sizes for outcomes in your derivation cohort), and since anticholinergic burden is mechanistically linked to decreased mobility, cognitive decline, and impaired global function, can the ACMI be used as a proxy or predictor for these outcomes in new populations or further validation is required or is this a limitation of the ACMI.<br><br>b) Clinical utility- used as a trigger tool for a SMR when a patient experience falls and delirium.	a) As described, in a separate external validation using UK Biobank and US All of Us data, the association between ACMI and cognitive impairment has been demonstrated. We are also undertaking further work to validate the ACMI for in-patient falls and delirium.<br><br>b) Our view is that the main ACMI use is proactive, to trigger an SMR in patients with high risk and high AC burden. However, we agree with the reviewer that there is potential for reactive use if a patient experiences a fall and/or delirium requiring hospitalisation and are actively exploring validation of the tool in secondary care as part of HDR UK and NIHR-funded work.	Discussion, Supplementary Box 2
Page 23, Lines 13 to 28 However, there are 7<br>….anticholinergic medications scoring 3 in the ACB scale that score 1 or less in the ACMI<br>(Amitriptyline, Chlorpromazine, Hydroxyzine, Imipramine, Nortriptyline, Promethazine, Tolterodine). This could represent an important contemporary reflection of the ACMI, as<br>some anticholinergic medications that have historically been prescribed in high dose (for example tricyclic antidepressants) are now prescribed at much lower dose for alternative<br>indications, typically management of neuropathic pain….<br><br>The above paragraph should be qualified with appropriate references as it can be deemed to be speculative as dose was never a consideration when the ACB scale was derived nor the ACMI.	We have altered the statement in question, and added some references<br><br>*"While neither the ACMI or the ACB incorporate dose, the lower ACMI weighting could represent more contemporary prescribing practices, as some anticholinergic medications that have historically been prescribed in high dose (for example tricyclic antidepressants) are now prescribed at much lower dose for alternative indications, typically management of neuropathic pain."*	

Clinical practitioners (e.g. pharmacists, general practitioners, advanced nurse practitioners) could implement the tool in primary care to automatically derive the ACMI<br>score based on their current prescriptions, along with their one year predicted risk of adverse outcomes, based on their age, sex, comorbidities and eFI score in addition to current prescriptions<br><br>It seems that the model has not yet been validated for other global clinical outcomes. Could the others please clarify how ACMI might be applied for 'adverse outcomes,' as this connection is not immediately clear to me?	We have altered the sentence to refer specifically to fall and delirium (rather than adverse outcomes)<br><br>*"The tool could be implemented in primary care to automatically derive the list of AC medications prescribed currently, ACMI score and the one year predicted risk of fall or delirium, for each patient aged >65 in a practice."*<br><br>As stated in response to previous reviewers, the ACMI has been validated to predict cognitive impairment using data from UK Biobank and the US All of Us study and we are undertaking further work to validate the ACMI in secondary care.	Discussion > pg 27
Table 3: Model coefficients and ACMI score …interpreting these coefficients as distinct and unbiased quantifications<br>of the relationship with the dependent variable.<br><br>I agree with the potential benefits discussed; however, I believe we should remain cautious regarding the scalability of ACMI, especially in light of the limitations I've highlighted previously. Importantly, the coefficients and ACMI score will change based on the dependent variable?	In our follow-on work validating the ACMI in secondary care, our intention is to maintain the current ACMI weighting, with the option of rescaling the score for the purpose of prediction of other outcomes.	
Example<br>A 70-year-old female patient with an eFI score of 0.25 (moderate frailty), with arthritis, for which she is prescribed codeine, arthritis treated<br>with long-term, low-dose maintenance prednisolone and atrial fibrillation for which she is anticoagulated with warfarin.<br>LP= 0.099 x (70-74) -0.001 x (70-74)2 + 3 x (0.25-0.0747) -0.097 -0.174 +<br>0.3344 + 0.080 -0.470 = -0.2127<br>One year risk = 2.9% x exp(-0.2127) = 2.3%<br><br>Thank you to the authors for providing this example. I think the authors should provide a stronger clinical context here to help interpret the findings. If the 2.3% figure they	We acknowledge that adding the number needed to treat would provide important clinical context. However, as outlined in our earlier response, the uncertainties in the trial evidence necessary to estimate NNT would be problematic.	

report is the adjusted annual risk, calculated from the baseline 2.9% using a hazard ratio of roughly 0.81 (since exp(-0.2127). Is it possible to derive NNT to prevent one hospital admission following a deprescribing intervention (SMR)-I am assuming such an intervention is low-cost and feasible. I could be entirely wrong in my argument but would welcome any clarification.		
**Reviewer 4**		
Comments:<br>The manuscript entitled: " Deriving the Anticholinergic Medication Index (ACMI): development and external validation of a risk prediction model for delirium and falls in older people" studied the development and validation of an index (ACMI) to predict the risk of delirium and falls in older adults from exposure to anticholinergic medications.<br><br>In my opinion, the study is interesting and its justification is adequate. A time-dependent Cox model was used, which allows for exposure to each medication change over time (month to month). This seems appropriate because many anticholinergic drugs are prescribed and discontinued throughout the year.<br><br>The model was adjusted for relevant clinical factors (age, sex, dementia, frailty, history of falls, etc.), making it clinically robust. Moreover, internal validation (cross-validation) and external validation (SAIL cohort, Wales) were performed, strengthening the methodological robustness.	Thank you	
- The model predicted the direction of risk well (C-index ~0.72), but the calibration was imperfect (calibration slope 0.69), meaning it tended to underestimate or overestimate in some groups.	We have added further detail regarding miscalibration in the discussion, in line with a previous reviewer's comment.	Discussion > pg 27
- Only "exposed/unexposed" was considered by month, without including dose or cumulative duration. This simplifies implementation in EHRs, but may reduce accuracy, given that anticholinergic toxicity	Please see our response to Other Editors (comment 1), who also commented on this limitation.	

is often dose-dependent.		
- PRN ("on-demand" medication) were excluded in the development cohort, but not in the validation cohort, which may have introduced heterogeneity and overestimated risk in the validation cohort.	Please see our response to 'Other Editors', comment 1.	
On the other hand, a composite outcome (delirium + falls) was used, which may mask differences in predictive ability for each event separately (e.g., the model predicted delirium better in Bradford and falls better in Wales). Respect this, could the authors explain why a composite outcome has been used?	Please see our response to Editors comments #3	
**General changes**		
In particular, please make sure you include the following:<br><br>1. What this paper adds/what is already known summary box, as described in our article preparation guidance<br>2. Name of the ethics committee or IRB, ID number of the approval, and a statement that participants gave informed consent before taking part, as per our research ethics guidance. If ethics committee approval was not required, please state so clearly and explain the reasons why<br>3. Patient confidentiality forms, when appropriate<br>4. Competing interests statement and ICMJE forms completed for all authors<br>5. Contributorship statement and guarantor<br>6. Transparency statement: Please include the following statement in the end matter of the paper: "The lead author (the manuscript's guarantor) affirms that the manuscript is an honest, accurate, and transparent account of the study being reported; that no important aspects of the study have been omitted; and that any discrepancies from the study as planned (and, if relevant, registered) have been explained."<br>7. The BMJ requires a data sharing statement for all research papers.	Thank you, we have made the relevant alterations throughout	

Additional requirements very depending on the type of research. Please check our data and code sharing policy carefully as we have different requirements depending on whether your article is reporting a clinical trial or not.<br>8. Funding statement and statement of the independence of researchers from funders<br>9. Patient and public involvement statement<br>10. Dissemination plans: At the end of the paper, please state how the results of your study have been, or will be, sent to patients and the public under the heading “Dissemination plans”. If you have prepared a lay summary, for example for your funders, please include it in a supplementary file. If you have not disseminated and have no plans to do so, please state why.<br>11. Reporting statement: We are not asking you to restructure the paper at this stage, but we do require the appropriate flow chart in the manuscript and the completed checklist uploaded as a supplemental file, with page numbers indicating where the questions are answered in your paper. Please note that some reporting statements have extensions in addition to the main statement, and we'll need to see the right one for your study design. They are all freely available at EQUATOR Network.<br>1. CONSORT statement for a randomised trial: Please note that there are several CONSORT extensions, for example for chart pragmatic trials or cluster randomised trials, in addition to the main statement, and we'll need to see the right one for your study design.<br>2. CONSORT abstract for a randomised trial: A structured abstract that follows the CONSORT guidance on RCT abstracts.<br>3. TIDieR checklist for reporting interventions in a randomised controlled trial.		

4. PRISMA statement for systematic reviews and meta-analyses.<br>5. STROBE statement for observational studies.<br>6. STARD statement for diagnostic studies.<br>7. CHEERS statement for economic evaluations.<br>12. Please ensure the paper complies with *The BMJ*'s style, as detailed below:<br>1. Title: This should include the study design, for example "systematic review and meta-analysis."<br>2. Please provide a structured abstract, with key summary statistics in the format described in our [article preparation guidance](). For every clinical trial, and for any other registered study, the last line of the abstract must list the study registration number and the name of the register.<br>1. Please report all outcomes that were listed in the trial registry, or explain that you will publish them elsewhere. Please clearly identify each outcome as primary, secondary, or post-hoc in the text, abstract, and any tables or figures. We expect authors to report prespecified outcomes. If outcomes in the trial registry have later been changed, please explain the reasons for the change and the dates of the change in the paper. You may report the changed outcomes, but we will expect you to also report on the originally specified outcomes unless otherwise agreed with the		

handling editor for your paper.<br>2. Occasionally the outcomes that are prespecified in a trial registry do not match up with those included in the trial protocol. When there are discrepancies between protocol and registry specified outcomes, we expect the paper to report and interpret the registry specified outcomes. You may also report any protocol specified outcomes, but if you do please be sure to include the date of the protocol and the point at which each outcome was added to the protocol, and explain why the registry entry differed from the protocol and why the registry was not updated to reflect any protocol changes.<br>3. Introduction: This should cover no more than three paragraphs, focusing on the research question and your reasons for asking it now.<br>4. Methods: For an intervention study, the manuscript should include enough information about the intervention(s) and comparator(s), even if this was usual care, for reviewers and readers to fully understand what happened in the study. To enable readers to replicate your work or implement the interventions in their own practice, please also provide any relevant detailed descriptions and materials, uploaded as one or more supplemental files, including video and audio		

files, where appropriate. Alternatively, please provide in the manuscript urls to openly accessible websites where these materials can be found.<br>5. Results: Please report statistical aspects of the study in line with the Statistical Analyses and Methods in the Published Literature (SAMPL) guidelines. Please include in the results section of your structured abstract (and, of course, in the article's results section) the following terms, as appropriate:<br>1. For a clinical trial: Absolute event rates among experimental and control groups; RRR (relative risk reduction); NNT or NNH (number needed to treat or harm) and its 95% confidence interval (or, if the trial is of a public health intervention, number helped per 1000 or 100,000.)<br>2. For a cohort study: Absolute event rates over time (eg 10 years) among exposed and non-exposed groups; RRR (relative risk reduction.)<br>3. For a case control study: OR (odds ratio) for strength of association between exposure and outcome.<br>4. For a study of a diagnostic test: Sensitivity and specificity; PPV and NPV (positive and negative predictive values.)<br>5. For a systematic review and/or meta-analysis: Point estimates and confidence intervals		

for the main results; one or more references for the statistical package(s) used to analyse the data, for example RevMan for a systematic review. There is no need to provide a formal reference for a very widely used package that will be very familiar to general readers, for example STATA, but please say in the text which version you used. For articles that include explicit statements of the quality of evidence and strength of recommendations, we prefer reporting using the GRADE system.<br>6. Discussion: To minimise the risk of careful explanation giving way to polemic, please write the discussion section of your paper in a structured way. Please follow this structure: i) statement of principal findings of the study; ii) strengths and weaknesses of the study; iii) strengths and weaknesses in relation to other studies, discussing important differences in results; iv) what your study adds (whenever possible, please discuss your study in the light of relevant systematic reviews and meta-analyses); v) meaning of the study, including possible explanations and implications for clinicians and policymakers and other researchers; vi) how your study could promote better decisions; vi) unanswered questions and future research.<br>7. Footnotes and statements: Please include relevant		

statements as referred to above.		

VERSION 2 – REVIEW

**REVIEWER NAME**	Perera, Rafael
**REVIEWER AFFILIATION**	University of Oxford, Primary Care Health Sciences
**REVIEWER CONFLICT OF INTEREST**	None
**DATE REVIEW RETURNED**	14-May-2026

**GENERAL COMMENTS**	Stats Review<br><br>Many thanks to the authors for their revised article, which adequately addresses all editors' and reviewers' questions. Apologies, as I have one more question/clarification. In the Discussion section, the authors comment on using their score to create a ranking based on individuals' risk within practice. This would be based on the risk prediction model (LP) that they have derived and validated. However, later in the manuscript, when presenting an example in Supplementary Box 2, they comment that 'In practice we recommend that the ACMI output (patient name, age, sex, eFI score, AC medications, ACMI score, and One year risk) is generated for all patients… and ordered by their ACMI score OR {my emphasis} their predicted risk…'<br><br>There is an issue here as the ranking obtained using the ACMI score (medication score) is NOT the same as the score obtained from the one-year predicted risk. Given the approach taken, the ACMI score (medication) is a transformation of some coefficients of the LP that estimates the risk of the (composite) outcome. The ACMI score (medication) is not an accurate representation of the risk; therefore, it should not be the basis for the ranking. A 'pure medication' score would require a different approach from the one used.<br><br>This also has implications for the text used in the manuscript, as the term ACMI score is used interchangeably to refer to both the medication score and the one-year prediction model. Please can the authors clarify the correct way to use their scores and, if they want to use them interchangeably, justify this?<br><br>Minor:<br>In the Example (Supplementary Box 1), the number for the linear predictor (LP) is incorrect. It has not been changed to -0.1917 in the calculation. It is correctly adjusted to calculate the One-year risk.<br><br>When presenting the 'Data sources', the numbers in the Connected Bradford of 800,000 people and then 600,000 people do not make sense. Please adjust the text.

VERSION 2 – AUTHOR RESPONSE

Many thanks to the authors for their revised article, which adequately addresses all editors' and reviewers' questions. Apologies, as I have one more question/clarification. In the Discussion section, the authors comment on using their score to create a ranking based on individuals' risk within practice. This would be based on the risk prediction model (LP) that they have derived and validated. However, later in the manuscript, when presenting an example in Supplementary Box 2, they comment that 'In practice we recommend that the ACMI output (patient name, age, sex, eFI score, AC medications, ACMI score, and One year risk) is generated for all patients… and ordered by their ACMI score OR {my emphasis} their predicted risk…'<br><br>There is an issue here as the ranking obtained using the ACMI score (medication score) is NOT the same as the score obtained from the one-year predicted risk. Given the approach taken, the ACMI score (medication) is a transformation of some coefficients of the LP that estimates the risk of the (composite) outcome. The ACMI score (medication) is not an accurate representation of the risk; therefore, it should not be the basis for the ranking. A 'pure medication' score would require a different approach from the one used.<br><br>This also has implications for the text used in the manuscript, as the term ACMI score is used interchangeably to refer to both the medication score and the one-year prediction model. Please can the authors clarify the correct way to use their scores and, if they want to use them interchangeably, justify this?	We recommend ranking patients using information from both ACMI and the clinical prediction model - this is what we have done in feasibility testing and it was supported by practitioners. The idea of using both is that it helps identify people who might be at high risk overall but are not taking multiple AC medications, so who would not be invited for SMR to target potentially modifiable medications.<br><br>We have altered the discussion to state "Therefore, we recommend using the ACMI score in parallel with overall risk of delirium or falls calculated from the CPM, to identify or prioritise patients with higher anticholinergic burden who are at greatest risk of adverse clinical outcomes.<br><br>We have updated the Supplementary Box 2 footer to say AND rather than OR<br><br>We have also referred to the clinical prediction model as CPM throughout to make a clearer distinction between the risk prediction model and the ACMI score.
Minor:<br>In the Example (Supplementary Box 1), the number for the linear predictor (LP) is incorrect. It has not been changed to -0.1917 in the calculation. It is correctly adjusted to calculate the One-year risk.	Thank you for spotting this, we have corrected this important error

When presenting the 'Data sources', the numbers in the Connected Bradford of 800,000 people and then 600,000 people do not make sense. Please adjust the text.	Thank you for spotting this, we have corrected the error
