# Supplementary material for: Deriving the anticholinergic medication index for delirium and falls in older people: development and external validation of a risk prediction model
Source: BMJ Med. 2026 Nov 4;5(1):e002835. doi: 10.1136/bmjmed-2026-002835 (PMC13735312; doi:10.1136/bmjmed-2026-002835)
Supplement: Supplementary file 2 [file bmjmed-5-1-s001.pdf]

Supplementary Box 1 Example calculation of one year predicted risk of fall or delirium and the ACMI score

Prediction model	ACMI score
1 year risk of hospital admission for falls or delirium = 1year baseline hazard x exp (LP)<br><br>Where 1 year baseline hazard is 2.9%, LP is the linear predictor and exp is the exponential function.<br><br>LP= 0.099 x (Age-74) -0.001 x $(Age-74)^2$ -0.097 (if female) + 3.12 x (eFI_score-0.0747) + 0.557 x (Memory or cognitive problems) -0.045 x (Visual impairment) -0.101 x (Hearing impairment) + 0.443 x (Parkinsonism or tremor) + 0.493 x (History of falls) -0.002 x (Urinary incontinence) -0.018 x (Osteoporosis) -0.174 x (Arthritis) -0.76 x (Alverine) + 1.806 x (Amantadine) + 0.573 x (Amisulpride) -0.071 x (Amitriptyline) + 0.625 x (Aripiprazole) -0.146 x (Atenolol) + 1.177 x (Atropine) + 0.335 x (Baclofen) -0.483 x (Bendroflumethiazide) -0.609 x (Betamethasone) + 0.458 x (Bumetanide) + 0.374 x (Buprenorphine) -0.598 x (Captopril) + 0.73 x (Carbamazepine) -0.144 x (Cetirizine) + 0.198 x (Chlorphenamine) -0.446 x (Chlorpromazine) + 0.543 x (Cimetidine) + 0.448 x (Cinnarizine) + 0.335 x (Citalopram) -0.107 x (Clomipramine) + 0.166 x (Clonazepam) -0.568 x (Clonidine) + 0.334 x (Codeine) -0.642 x (Colchicine) + 0.897 x (Cyclizine) + 0.454 x (Darifenacin) -1.309 x (Desloratadine) + 0.109 x (Dexamethasone) + 0.338 x (Diazepam) + 0.117 x (Digoxin) -0.17 x (Dihydrocodeine) -0.868 x (Dipyridamole) -0.366 x (Dosulepin) -0.174 x (Doxazosin) + 0.735 x (Escitalopram) + 0.549 x (Fentanyl) + 0.767 x (Fesoterodine) + 0.034 x (Fexofenadine) + 0.552 x (Fluoxetine) + 0.086 x (Flupenthixol) + 0.18 x (Furosemide) -0.192 x (Glycopyrronium bromide) + 0.604 x (Haloperidol) + 0.617 x (Hydralazine) -0.266 x (Hydrochlorothiazide) + 0.3 x (Hydrocortisone) -0.386 x (Hydroxyzine) + 0.293 x (Hyoscine) -0.282 x (Imipramine) -0.447 x (Indapamide) + 0.591 x (Indoramin) + 0.654 x (Ipratropium) + 0.044 x (Isosorbide) -0.102 x (Loperamide) -1.07 x (Loratadine) + 0.331 x (Lorazepam) -0.07 x (Mebeverine) + 0.223 x (Metoprolol) + 1.081 x (Midazolam) + 0.367 x (Mirtazapine) + 0.599 x (Morphine) + 0.137 x (Nefopam) + 0.161 x (Nifedipine) -0.063 x (Nortriptyline) + 0.661 x (Olanzapine) + 0.231 x (Oxybutynin) + 0.63 x (Oxycodone) + 0.213 x (Paroxetine) + 0.08 x (Prednisolone) -0.025 x (Prochlorperazine) + 0.665 x (Procyclidine) -0.158 x (Promethazine) + 0.378 x (Quetiapine) + 0.394 x (Ranitidine) + 0.39 x (Risperidone) -0.246 x (Solifenacin) + 0.289 x (Temazepam) -0.015 x (Theophylline) + 0.301 x (Tiotropium bromide) + 0.014 x (Tolterodine) + 0.46 x (Tramadol) + 0.015 x (Trazodone) + 0.919 x (Trihexyphenidyl) + 0.385 x (Trospium chloride) + 0.27 x (Umeclidinium) + 0.409 x (Venlafaxine) -0.47 x (Warfarin)	0.5 (if Alverine) + 2.3 (if Amantadine) + 1.4 (if Amisulpride) + 1 (if Amitriptyline) + 1.5 (if Aripiprazole) + 0.9 (if Atenolol) + 1.9 (if Atropine) + 1.3 (if Baclofen) + 0.7 (if Bendroflumethiazide) + 0.6 (if Betamethasone) + 1.4 (if Bumetanide) + 1.3 (if Buprenorphine) + 0.6 (if Captopril) + 1.5 (if Carbamazepine) + 0.9 (if Cetirizine) + 1.2 (if Chlorphenamine) + 0.7 (if Chlorpromazine) + 1.4 (if Cimetidine) + 1.3 (if Cinnarizine) + 1.3 (if Citalopram) + 0.9 (if Clomipramine) + 1.1 (if Clonazepam) + 0.6 (if Clonidine) + 1.3 (if Codeine) + 0.6 (if Colchicine) + 1.7 (if Cyclizine) + 1.3 (if Darifenacin) + 0.1 (if Desloratadine) + 1.1 (if Dexamethasone) + 1.3 (if Diazepam) + 1.1 (if Digoxin) + 0.9 (if Dihydrocodeine) + 0.4 (if Dipyridamole) + 0.8 (if Dosulepin) + 0.9 (if Doxazosin) + 1.6 (if Escitalopram) + 1.4 (if Fentanyl) + 1.6 (if Fesoterodine) + 1 (if Fexofenadine) + 1.4 (if Fluoxetine) + 1.1 (if Flupenthixol) + 1.1 (if Furosemide) + 0.9 (if Glycopyrronium bromide) + 1.5 (if Haloperidol) + 1.5 (if Hydralazine) + 0.8 (if Hydrochlorothiazide) + 1.2 (if Hydrocortisone) + 0.7 (if Hydroxyzine) + 1.2 (if Hyoscine) + 0.8 (if Imipramine) + 0.7 (if Indapamide) + 1.4 (if Indoramin) + 1.5 (if Ipratropium) + 1 (if Isosorbide) + 0.9 (if Loperamide) + 0.2 (if Loratadine) + 1.3 (if Lorazepam) + 1 (if Mebeverine) + 1.2 (if Metoprolol) + 1.8 (if Midazolam) + 1.3 (if Mirtazapine) + 1.5 (if Morphine) + 1.1 (if Nefopam) + 1.1 (if Nifedipine) + 1 (if Nortriptyline) + 1.5 (if Olanzapine) + 1.2 (if Oxybutynin) + 1.5 (if Oxycodone) + 1.2 (if Paroxetine) + 1.1 (if Prednisolone) + 1 (if Prochlorperazine) + 1.5 (if Procyclidine) + 0.9 (if Promethazine) + 1.3 (if Quetiapine) + 1.3 (if Ranitidine) + 1.3 (if Risperidone) +0.8 (if Solifenacin) + 1.2 (if Temazepam) + 1 (if Theophylline) + 1.2 (if Tiotropium bromide) + 1 (if Tolterodine) + 1.4 (if Tramadol) + 1 (if Trazodone) + 1.7 (if Trihexyphenidyl) + 1.3 (if Trospium chloride) + 1.2 (if Umeclidinium) + 1.3 (if Venlafaxine) + 0.7 (if Warfarin) +

**Example**<br>Mrs Susan Smith is a 70-year-old female patient with an eFI score of 0.25 (moderate frailty), with arthritis, for which she is prescribed codeine, arthritis treated with long-term, low-dose maintenance prednisolone and atrial fibrillation for which she is anticoagulated with warfarin.	
LP= 0.099 x (70-74) -0.001 x $(70-74)^2$ + 3.12 x (0.25-0.0747) -0.097 -0.174 + 0.3344 + 0.080 -0.470 = -0.2127<br><br>One year risk = 2.9% x exp(-0.2127) = 2.3%	ACMI score = 1.3 + 1.1 + 0.7 = 3.1

The ACMI should be used in conjunction with clinical judgment to assess anticholinergic burden. Some medications may be prescribed pro re nata (PRN); in these cases the exposure status of patients prescribed these medications may be uncertain and their ACMI score may actually be inflated. We advise clinicians to be aware that some medications in practice may be prescribed as PRNs and so there is less certainty regarding the patients exposure status to these medications. We advise avoiding interpreting these coefficients as distinct and unbiased quantifications of the relationship with the dependent variable.
