# Supplementary material for: Deriving the anticholinergic medication index for delirium and falls in older people: development and external validation of a risk prediction model
Source: BMJ Med. 2026 Nov 4;5(1):e002835. doi: 10.1136/bmjmed-2026-002835 (PMC13735312; doi:10.1136/bmjmed-2026-002835)
Supplement: Supplementary file 3 [file bmjmed-5-1-s002.pdf]

Supplementary Box 2 Example ACMI output in primary care practices

Patient name	Age	Sex	eFI	AC medications	ACMI score	One year predicted risk
Mrs Susan Smith	70	F	0.25	Codeine, Prednisolone, Warfarin	3.1	2.3%
Mr John Brown	66	M	0.06	Warfarin, Venlafaxine	2.0	1.1%
Ms Sarah Johnson	85	F	0.39	Oxybutynin, Amitriptyline, Hydroxyzine, Trihexyphenidyl	4.6	66.4%
Mr Marcus Evans	90	M	0.08	Alverine, Warfarin	1.2	10.6%

In practice we recommend that the ACMI output (i.e. patient name, age, sex, eFI score, AC medications, ACMI score and One year risk) is generated for all patients aged >65 in a practice and the patients ordered by their ACMI score or their predicted risk, to help practices prioritise patients to invite for SMR.
