# Supplementary material for: Deriving the anticholinergic medication index for delirium and falls in older people: development and external validation of a risk prediction model
Source: BMJ Med. 2026 Nov 4;5(1):e002835. doi: 10.1136/bmjmed-2026-002835 (PMC13735312; doi:10.1136/bmjmed-2026-002835)
Supplement: Supplementary file 4 [file bmjmed-5-1-s004.pdf]

Supplementary Table 1 AC medications in the ACB, with scores, relative to those in the ACB

Medication Name	ACB Score (1-3)	ACMI (0.1-2.3)
Alimemazine	1	-
Alverine	1	0.5
Alprazolam	1	-
Atenolol	1	0.9
Brompheniramine maleate	1	-
Bupropion hydrochloride	1	-
Captopril	1	0.6
Chlorthalidone	1	-
Cimetidine hydrochloride	1	1.4
Ranitidine	1	-
Clorazepate	1	-
Codeine	1	1.3
Colchicine	1	0.6
Coumadin	1	0.7
Diazepam	1	1.3
Digoxin	1	1.1
Dipyridamole	1	0.4
Disopyramide phosphate	1	-
Fentanyl	1	1.4
Furosemide	1	1.1
Fluvoxamine	1	-
Haloperidol	1	1.5
Hydralazine	1	1.5
Hydrocortisone	1	1.2
Isosorbide	1	1
Loperamide	1	0.9
Metoprolol	1	1.5
Morphine	1	1.5
Nifedipine	1	1.1
Prednisolone	1	1.1
Quinidine	1	-
Risperidone	1	1.3
Theophylline	1	1
Trazodone	1	1
Triamterene	1	-
Amantadine	2	2.3
Belladone alkaloids	2	-
Carbamazepine	2	1.5
Cyclobenzaprine	2	-
Cyproheptadine	2	-
Empracet	2	-
Loxapine	2	-
Meperidine	2	-

Methotrimeprazine	2	-
Molindone	2	-
Oxcarbazepine	2	-
Pethidine hydrochloride	2	-
Pimozide	2	-
Amitriptyline	3	1
Amoxapine	3	-
Atropine	3	1.9
Benztropine	3	-
Brompheniramine	3	-
Carbinoxamine	3	-
Chlorpheniramine	3	1.2
Chlorpromazine	3	0.7
Clemastine	3	-
Clomipramine	3	0.9
Clozapine	3	-
Darifenacin	3	1.3
Desipramine	3	-
Dicyclomine	3	-
Dimenhydrinate	3	-
Diphenhydramine	3	-
Doxepin	3	-
Flavoxate	3	-
Hydroxyzine	3	0.7
Hyoscyamine	3	1.2
Imipramine	3	0.8
Meclizine	3	-
Nortriptyline	3	1
Olanzapine	3	1.5
Orphenadrine	3	-
Oxybutynin	3	1.2
Paroxetine	3	1.2
Perphenazine	3	-
Procyclidine	3	1.5
Promazine	3	-
Promethazine	3	0.9
Propentheline	3	-
Pyrilamine	3	-
Quetiapine	3	1.3
Scopolamine	3	-
Thioridazine	3	-
Tolterodine	3	1
Trifluoperazine	3	-
Trihexyphenidy	3	1.7
Trimipramine	3	-
